# Local electronic structure rearrangements and strong anharmonicity in YH_3_ under pressures up to 180 GPa

**DOI:** 10.1038/s41467-021-21991-x

**Published:** 2021-03-19

**Authors:** J. Purans, A. P. Menushenkov, S. P. Besedin, A. A. Ivanov, V. S. Minkov, I. Pudza, A. Kuzmin, K. V. Klementiev, S. Pascarelli, O. Mathon, A. D. Rosa, T. Irifune, M. I. Eremets

**Affiliations:** 1 Institute of Solid State Physics University of Latvia, Riga, LV-1063 Latvia; 2 National Research Nuclear University MEPhI (Moscow Engineering Physics Institute), Moscow, 115409 Russia; 3 Max-Planck Institut für Chemie, PO Box 3060, Mainz, DE-55128 Germany; 4 MAX IV Laboratory, PO Box 118, Lund, SE-221 00 Sweden; 5 European Synchrotron Radiation Facility, BP 220, Grenoble, F-38043 France; 6 European X-Ray Free Electron Laser (XFEL) GmbH, Schenefeld, 22869 Germany; 7 Geodynamics Research Center, Ehime University, Matsuyama, 790-8577 Japan

**Keywords:** Electronic properties and materials, Superconducting properties and materials

## Abstract

The discovery of superconductivity above 250 K at high pressure in LaH_10_ and the prediction of overcoming the room temperature threshold for superconductivity in YH_10_ urge for a better understanding of hydrogen interaction mechanisms with the heavy atom sublattice in metal hydrides under high pressure at the atomic scale. Here we use locally sensitive X-ray absorption fine structure spectroscopy (XAFS) to get insight into the nature of phase transitions and the rearrangements of local electronic and crystal structure in archetypal metal hydride YH_3_ under pressure up to 180 GPa. The combination of the experimental methods allowed us to implement a multiscale length study of YH_3_: XAFS (short-range), Raman scattering (medium-range) and XRD (long-range). XANES data evidence a strong effect of hydrogen on the density of 4*d* yttrium states that increases with pressure and EXAFS data evidence a strong anharmonicity, manifested as yttrium atom vibrations in a double-well potential.

## Abstract (web summary)

The discovery of superconductivity in metal hydrides requires an understanding of hydrogen interactions with the heavy atom sublattice under high pressure. Here, the authors report evidence of a strong effect of hydrogen on the density increase of 4*d* yttrium states and strong anharmonic vibrations of yttrium atoms in YH_3_.

## Introduction

In recent years, a breakthrough in the field of high-temperature superconductivity (SC) has been achieved due to the discovery of a new class of materials, where the critical temperature for superconductivity (*T*_*c*_) exceeds 200 K. This includes the so-called superhydrides: LaH_10_ with *T*_*c*_ ≈ 250 K^1,2^, YH_9_ with *T*_*c*_ ≈ 241 K^3^, YH_6_ with *T*_*c*_ ~ 220 K^3,4^ as well as SH_3_ where *T*_*c*_ = 203 K^5^. These are phonon-mediated superconductors, where the mechanisms determining superconductivity are conceptually well understood. Furthermore, there is a possibility that room and even higher *T*_*c*_ can be reached^6,7^ (during the review process of this paper, achieving *T*_*c*_ = 285 K at *P* ~ 270 GPa in the ternary C-S-H-system has been reported^8^, see also comments by Lv et al.^9^). However, this progress has been achieved at the cost of the application of very high pressure. Typically, some 150-200 GPa are required, which can be attained by the diamond anvil cell (DAC) technique. These findings, together with advances in development of computational methods (see Flores-Livas^10^ for the review), prove the concept that high *T*_*c*_ can be attained in systems containing light elements (which promote high frequency of the lattice vibrations)—hydrogen being an extreme case^11^. However, it is unclear how hydrogen under high pressure interacts with the sublattice of heavy atoms in metal hydrides at the atomic scale.

Here, we address this fundamental but open question of how hydrogen affects and reconstructs the yttium ionicity and local crystal structure under high pressure through a study of stoichiometric YH_3_ as an archetypal metal hydride. This composition is of particular interest for several reasons. First, it has as much hydrogen (in atomic percentage) as SH_3_ does—a superconductor with an extremely high critical temperature of 200 K, remaining stable up to 400 GPa (the highest studied)^12^. Second, at ambient pressure, a transition from metallic to insulating state occurs on a change of the composition from YH_2_ to YH_3_ with concomitant structural transition from fcc to hcp arrangement of Y atoms^13^. Under compression, the compound transforms back to fcc metal at pressures of 8–20 GPa^14^. The high-pressure fcc phase, which has hydrogen located in the octahedral and tetrahedral interstices ($Fm\bar{3}m$ space group), is predicted to become a superconductor with T_*c*_ = 40 K at *P* = 17.7 GPa, which is the record low external pressure^15^ for predicting superconductivity in hydrides^16^.

However, superconductivity has not been observed experimentally^3,17^ (moreover, at 18 GPa the material does not even adopt the expected fcc structure).

Several questions also arise concerning how the pressure-induced insulator-metal (IM) transition occurs. While infrared (IR) transmission spectrum collapses at 23.5  GPa^18^, electrical resistivity gradually decreases and only reaches values typical for a metallic state at ~40 GPa^17^. The structural hcp → fcc phase transition, although falling within this range, does not seem to have an evident correlation with the IM transition. It also appears to be sluggish with an intermediate state spanning the range from 8 to 20 GPa according to XRD data^14,19,20^. The Raman signal that is observed above 20 GPa^21,22^ contradicts the centrosymmetric $Fm\bar{3}m$ structure observed by XRD and predicted in the calculations^16^. Similarly, for the hcp structure the Raman measurements at ambient pressure^23^ exclude the existence of a centrosymmetric $P\bar{3}c1$ structure reported from neutron diffraction studies^24,25^. Several models have been proposed to reconcile this contradiction, which assume local distortions of the crystal lattice. Meanwhile, both the nature of the insulating state and its exact structure, including the hydrogen subsystem, are still under debate^25,26^.

We used locally sensitive X-ray absorption fine structure spectroscopy (XAFS) to clarify the features of the phase transitions in YH_3_ under pressure and, most importantly, understand the hydrogen influence on the local electronic and spatial structure rearrangement under high pressure.

Accurate determination of the hydrogen positions in hydrides using the experimental XAFS (EXAFS - extended X-ray absorption fine structure + XANES - X-ray absorption near edge structure) technique is a challenging task because the photoelectron scattering amplitude for hydrogen is too small. Nonetheless, hydrogen affects the structure, leading to lattice rearrangements that can be detected by EXAFS as a variation of the local interatomic distances between metal atoms^27–30^ or changes in the local density of unoccupied states that can be probed by XANES^31–33^. However, XAFS has not been used to study hydrides at ultrahigh pressures until recently because strong Bragg diffraction originating from the conventional single crystal diamond anvil cells (DAC) that are used to generate pressure introduces spike-like peaks called “glitches” into EXAFS spectra, making the latter almost impossible to analyze. However, the appearance of Nano Polycrystalline Diamond (NPD) anvils in the field of high-pressure EXAFS has revolutionized the activity with a tremendous increase in the data quality, resulting in glitch-free spectra^34,35^. The advantage of NPD anvils has since been confirmed in high-pressure XAFS studies of different compounds in a number of works^36–38^ and the highest pressure above 300 GPa was achieved in the Re metal studies^39^. However, until now, there have been no attempts at XAFS studies of hydrogen-containing compounds under pressure. We fill this gap and present the high pressure XAFS data of the YH_3_ hydride obtained using nanodiamond anvils^40,41^.

YH_3_ was the best option for the first XAFS studies among the metal hydrides considering that at the Y *K*-edge (17,038 eV) the absorption of diamond anvils can be neglected. At that moment, LaH_10_, the highest-temperature superconductor, was not optimal because of strong X-ray absorption in diamond at the La *L*_3_-edge (5483  eV). In addition, the synthesis of LaH_10_ required high pressure of hydrogen at high temperature^1^, such as of YH_6_ and YH_9_^3^, which may be accompanied by strong diffusion of hydrogen into nano-pores of nano-diamonds^42^. In the future, we plan to overcome these problems by using pulsed laser heating in the synthesis process, and we hope that an increase in X-ray intensity by more than an order of magnitude after the European Synchrotron Radiation Facility (ESRF) upgrade in 2020 will allow us to measure *L*_3_-La XANES spectra.

Thus, the goal of this study was to get insight into the nature of the hcp-fcc phase transition and to clarify the role of hydrogen in the local electronic and crystal structure rearrangements in YH_3_, including cardinal yttrium charge changes, under pressure up to 180 GPa using XAFS spectroscopy, combined with X-ray diffraction (XRD) and supplemented with resistivity and Raman spectra measurements.

This combination of experimental methods have allowed us to implement a multiscale length study of YH_3_: XAFS (short range), Raman scattering (medium range), and XRD (long range). New Raman scattering and resistivity measurements allowed us to extend the previously available data, which covered a limited pressure range up to 40 GPa^14,19–21^. These new data complemented our careful X-ray (XRD, EXAFS, XANES) experiments, and lead to a deeper understanding of the effect of strong anharmonicity and local structure distortions in YH_3_ under pressure.

## Results

**Fig. 1:**
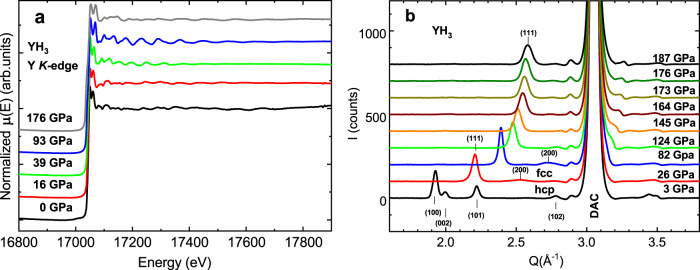
Pressure-dependent XAS and XRD of YH_3_. **a** The normalized X-ray absorption spectra of YH_3_, measured above the Y *K*-edge at different pressures up to 176 GPa; **b** The pressure dependence of the X-ray diffraction patterns for YH_3_.

We undertook two pressure runs, S1 and S2, using anvils with culet size 60 and 150 μm, in which XAFS spectra, combined with XRD, were measured. Pressures of 176 GPa and 93 GPa have been achieved, respectively (see the samples description in the Methods section). We obtained glitch-free X-ray absorption spectra (XAS) at the Y *K*-edge for YH_3_ with high signal-to-noise ratio up to 16.5 Å^−1^ in momentum space. The XAS spectra of the S1 and S2 samples were normalized following the conventional procedure, as presented in Fig. 1a (the initial spectra are shown in the Supplementary Note 1).

Our XRD data (see Fig. 1b and Supplementary Note 2), which were obtained concomitantly with the XAFS measurements, confirm the previously reported hcp → fcc phase transition with an intermediate state between 8 and 20 GPa^14,19,20^. In addition, the Raman data (see Supplementary Note 3) can be interpreted as the appearance of intermediate *C*2/*m*-type structure in the range 20–40 GPa attributed to the consecutive Peierls distortions^21^ in accordance with the theoretical prediction^43^.

**Fig. 2:**
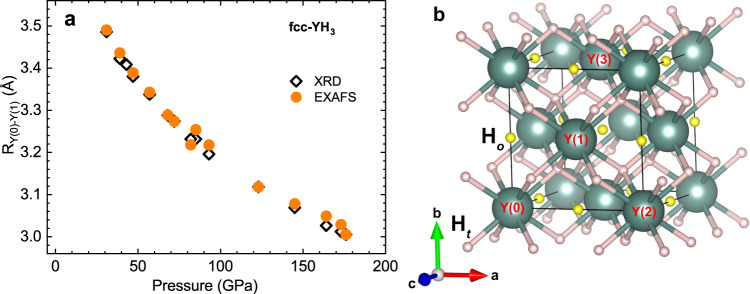
The crystal structure of YH_3_. **a** Pressure dependence of the interatomic distance $R(\,\text{Y(0)-Y(1)}\,)=a/\sqrt{2}$, (*a* is the lattice parameter) for fcc YH_3_ obtained from XRD versus Y *K*-edge EXAFS data; **b** Crystal structure of fcc YH_3_ phase. Y(0) and Y(2) denote yttrium atoms in the corners of the cube, while Y(1) atoms are in the centers of nearest faces and Y(3) atoms are in the centers of the neighbor faces. Small white balls mark hydrogen atoms H_*t*_ in tetrahedral positions and yellow ones - H_*o*_ in octahedral positions.

The pressure dependence of the lattice parameter for fcc YH_3_ structure, obtained from the combined XRD and EXAFS data, is presented in Fig. 2a. The pressure dependences of the lattice parameter obtained by these independent methods, sensitive for the long range scale and the local scale, are in good agreement. The crystal structure is shown in Fig. 2b, where the X-ray absorbing yttrium atom is pointed as Y(0).

### XANES results

**Fig. 3:**
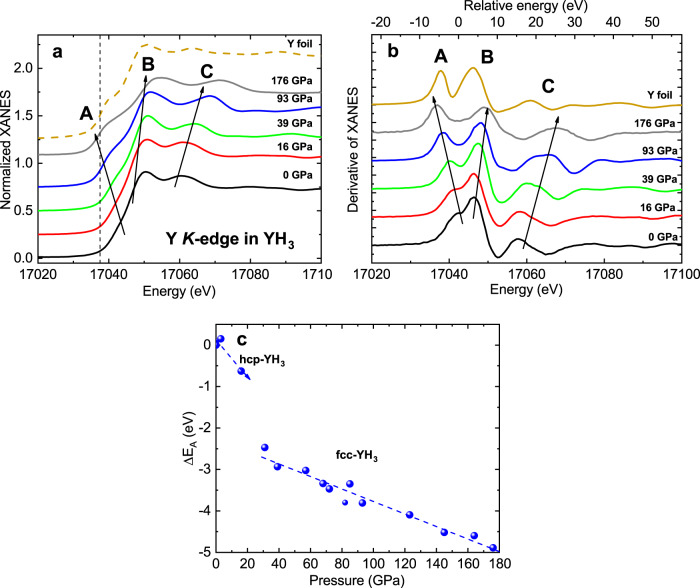
Pressure-dependent XANES spectra of YH_3_. **a** Pressure-dependent Y *K*-edge XANES spectra of YH_3_. The XANES of yttrium foil at normal pressure (dashed line) is shown for comparison, and the position of its edge is indicated by the vertical dashed line. **b** The first derivative of the normalized XANES spectra shown in **a**; **c** Pressure dependence of the Y *K*-edge shift ΔE_*A*_ relative to its position in the hcp phase.

The experimental Y *K*-edge XANES spectra of YH_3_ (samples S1 and S2) measured at pressures up to 176 GPa are compared in Fig. 3a with the XANES spectrum of yttrium foil. The impact of pressure can be seen in both the XANES spectra and their first derivative, which exhibit three main features that are denoted as **A**, **B**, **C** in Fig. 3b.

The X-ray absorption cross-section at the Y *K*-edge arises from the electric dipole transitions 1*s* → 5*p*,n*p*^44^. The feature **B** is attributed to the multiple scattering contribution and shows a small energy shift of ~2.5 eV to higher energies which is typical for other compounds under pressure^35^ and attributed to a decrease in the lattice parameter. The feature **C** arises from the appearance of the first EXAFS oscillation combined with multiple scattering features and shifts with pressure to higher energies in accordance with the increase in the EXAFS frequency at the lattice compression.

Our full-multiple-scattering calculations of XANES for yttrium metal foil are presented in Supplementary Note 4 and suggest that yttrium atoms, which are located in the first coordination shell of the absorber, produce the main contribution to the experimental XANES. In addition, the analysis of partial densities of states (DOS) indicates that the shoulder located at the absorption edge ~17040 eV can be ascribed to the transition to the hybridized state composed of *p*(Y) and *d*(Y) orbitals. Upon compression, the ionicity of YH_3_ is decreased and a significant amount of electronic charge is transferred back to the Y atoms^43^.

Thus, we attribute the shift of feature **A** of the yttrium absorption edge (E_*A*_) on the absolute energy scale (Fig. 3a, b) to a change of the effective charge of yttrium ions in YH_3_. The edge position shifts to the lower energies by ~5.5 eV upon increasing pressure, which suggests a decrease of the yttrium ion charge. Note that the edge position also changes abruptly upon phase transition from hcp to fcc YH_3_ phase ~20–30 GPa (Fig. 3c). This can be related to the transition at high pressure to a more conductive state with de-localized electrons (i.e., the insulator-to-metal transition), which efficiently screens the positive charge of the 1*s*(Y) core-hole during the photoabsorption process and thus reduces the electron binding energy at high pressure.

This large energy shift of the yttrium absorption edge is observed for the first time. It can be presumably related to the cardinal change of the density of 4*d*(Y)-state from 4*d*^0^ to 4*d*^1^, due to the approach of the hydrogen atom H_*o*_ to yttrium atom upon compression. Thus, we believe that Y(4*d*^1^)H_*o*_ complexes are formed at high pressure by analogy with LaH_3_^32^, where hydrogen atom captures an electron from La ion and forms a two-electron singlet states as an anion H^−^.

### Resistance and Raman scattering data

**Fig. 4:**
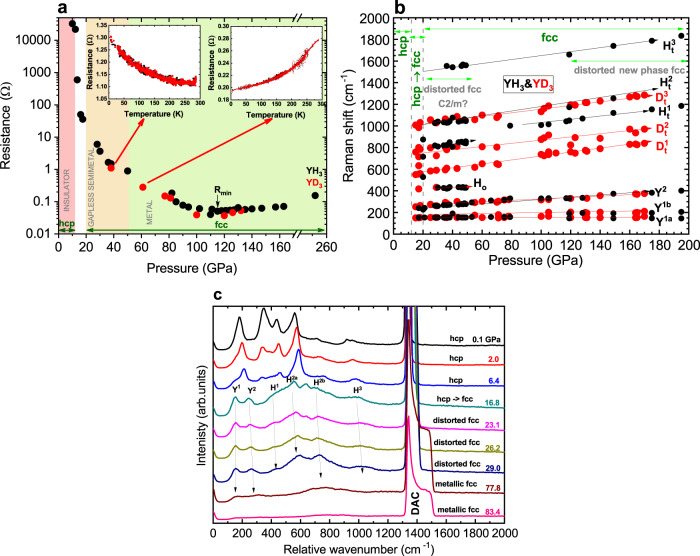
Resistance and Raman scattering. **a** Resistance versus pressure for YH_3_ (black dots) and YD_3_ (red dots). Insets show the temperature dependence of resistance at 38 and 61 GPa; **b** The dependence of Raman modes shift on pressure for YH_3_ and YD_3_; **c** The pressure dependence (up to 83.4 GPa) of the Raman scattering in YH_3_.

Several separate pressure runs were undertaken where resistance as a function of pressure and temperature was measured for both YH_3_ and YD_3_. Both isotopes behave identically. Similar to previous data^17^, at room temperature we observe about four orders in magnitude drop in resistance between ~10 and ~40 GPa. However, we do not confirm the maximum in R(T) at 50 GPa, reported in Matsuoka et al.^17^. From our data, resistance monotonically decreases until it reaches a minimum ~100–120 GPa, it then slightly increases up to the highest pressure studied (Fig. 4a). The semimetal-type behavior of resistance as a function of temperature holds until ~40 GPa, which is followed by a transition region spanning over 20 GPa where the resistance-versus-temperature curves exhibit random not reproducible behavior similar to that observed in Matsuoka et al.^17^. The metallic-type behavior appears above ~50 GPa (Fig. 4a, insets). We have not found superconductivity in YH_3_ at pressures up to 180 GPa and temperatures down to 5 K, which agrees with the results by Kong et al.^3^.

In the Raman scattering studies, we observed a monotonic shift of the Raman modes up to pressures of 190 GPa (see Fig. 4b). The intensity of the peaks in the Raman spectra decreases monotonically, and their width increases with an increase in pressure to 80 GPa (Fig. 4c). In the range of 80–100 GPa, the spectral curves are almost completely smoothed out, but at pressures above 110 GPa the Raman modes emerge again as pronounced narrow intense peaks in the spectra. Additional information concerning Raman scattering is presented in Supplementary Note 3.

### EXAFS results

**Fig. 5:**
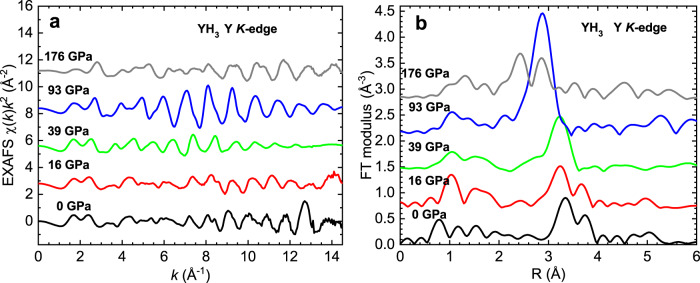
Pressure-dependent EXAFS spectra of YH_3_. **a** EXAFS spectra *χ*(*k*)*k*^2^ of YH_3_ at the *K*-edge of Y, and **b** their Fourier transform moduli at different pressures up to 176 GPa. The FTs represent raw experimental data without corrections for the scattering phase shifts due to the photoelectron backscattering.

Some typical EXAFS functions *χ*(*k*)*k*^2^ and their Fourier transform (FT) moduli for YH_3_ samples S1 and S2 at room temperature at different pressures are shown in Fig. 5.

The Fourier transform undergoes significant changes during the phase transition from the hcp to the fcc phase at ~20 GPa (compare curves at 16 and 39 GPa). The main FT peak at 2–4 Å, corresponding to the first yttrium Y(0)-Y(1) shell, is split into two peaks in the hcp phase (0 and 16 GPa) but merges into one peak in the fcc phase (39 and 93 GPa). The pressure range from 31 to 93 GPa is characterized by the strong increase in the amplitude of the Y-Y FT peak and its shift to shorter distances. As the pressure increases from 110 to 176 GPa, the Y-Y FT peak splits again into two peaks at shorter distances with considerably smaller amplitudes while remaining in the fcc phase according to the XRD data.

**Fig. 6:**
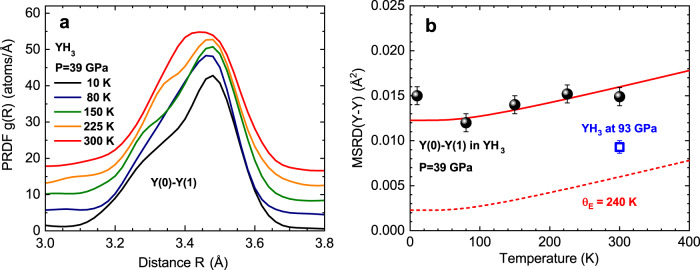
Temperature-dependent studies. **a** The pair radial distribution functions (PRDF) for YH_3_ in fcc phase at 39 GPa obtained from RMC simulations, and **b** the temperature dependence of the MSRD for the Y–Y bond in the first Y(0)–Y(1) coordination shell around Y(0). The dashed curve shows the Einstein harmonic model for the Y–Y atom pair with Einstein’s characteristic temperature Θ_*E*_ = 240 K. Meanwhile, the solid curve represents the Einstein model, shifted due to the static disorder contribution. The MSRD (blue open square) for the first yttrium shell in fcc YH_3_ phase at 93 GPa and 300 K is shown for comparison.

In addition to the RT measurements, we studied a temperature effect in the fcc phase at 39 GPa by cooling the sample down to 10 K. The use of the advanced analysis approach based on the reverse Monte-Carlo (RMC) method^45^ with an evolutionary algorithm (EA)^46^ (Supplementary Note 5) allowed us to extract the temperature dependences of the pair radial distribution function (PRDF) *g*_Y-Y_(*R*) and the mean square relative displacement (MSRD) *σ*^2^(Y-Y) for the first nearest Y(0)-Y(1) coordination shell (Fig. 6). In addition, the MSRD (blue open square) for the first yttrium shell in fcc YH_3_ phase at 93 GPa and 300 K is shown for comparison in Fig. 6b. It is worth noting here that the MSRD of the first Y-Y shell for 93 GPa is essentially less than for 39 GPa, which points to a more ordered structure.

As one can see from Fig. 6a, b, the amplitude of the PRDF increases while the MSRD of the first Y(0)-Y(1) shell decreases when the temperature decreases from 300 to 80 K. The MSRD behavior can be described by the Einstein harmonic model^47^ as ${\sigma }^{2}={\sigma }_{stat}^{2}+\frac{{\hslash }^{2}}{2{k}_{B}\mu }\frac{1}{{{{\Theta }}}_{E}}\coth \big[\frac{{{{\Theta }}}_{E}}{2T}\big]$, where ${\sigma }_{{\mathrm{stat}}}^{2}$ is a contribution of static disorder, Θ_*E*_ denotes Einstein’s characteristic temperature, *k*_*B*_ denotes Boltzmann constant, and *μ* denotes the reduced mass of the Y-Y atom pair.

However, at 10 K, the MSRD experiences a sharp jump from the harmonic curve by ~0.003 Å^2^, even exceeding the amplitude of thermal vibrations ${\sigma }_{d}^{2}$ at 300 K. This points to an anomalous increase in the amplitude of thermal vibrations with a decrease in temperature. This low-temperature anomaly in the temperature dependence of MSRD indicates strong anharmonicity, similar to that observed earlier in high temperature superconducting cuprates^48–50^, BaBiO_3_-based superconducting oxides^51,52^, and Fe-based HTSC^53^, which was successfully explained by vibration of oxygen ions in a double-well potential.

**Fig. 7:**
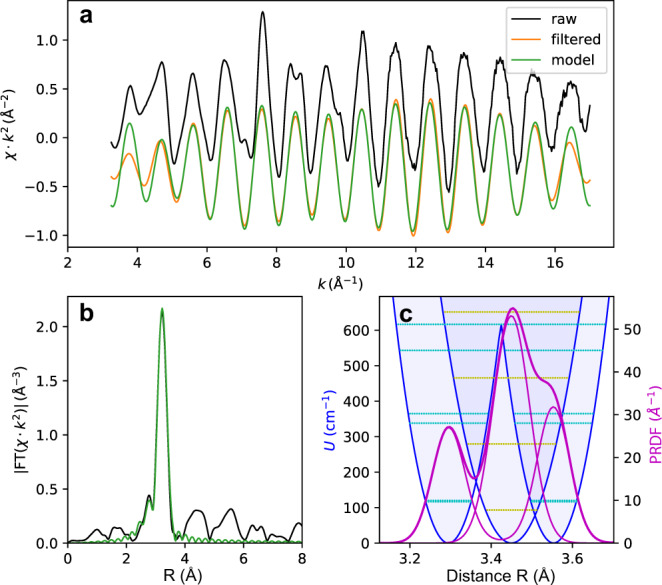
Strong anharmonicity in YH_3_. The results of the Y *K*-edge EXAFS spectra treatments for YH_3_ at 39 GPa and 10 K. **a** The raw experimental (black) and filtered (red) EXAFS function *χ*(*k*)*k*^2^ after back Fourier transform (BFT) in the real-space range (2.2–3.8 Å) with the model (green); **b** The Fourier transform (FT) modulus of EXAFS function *χ*(*k*)*k*^2^ (black) and the result of fitting (green) of the first Y(0)-Y(1) shell in the real-space range (2.2–3.8 Å); **c** The double-well and single-well potentials (blue solid curves) of Y atom vibrations and their PRDF’s (dark magenta) for the first Y(0)–Y(1) shell. The resulting PRDF is shown in bold dark magenta curves, the energy levels for the single-well potential are yellow, and the energy levels for the double-well one are dark cyan.

To take an anharmonicity into account, we treated the Y *K*-edge EXAFS spectra of YH_3_ for 39 GPa at 10 K in real space in the strong anharmonicity approximation, as in Menushenkov and Klementev^52^ (see Supplementary Note 6) using the VIPER program^54^, and we obtained the shape and the parameters of the potential for Y atom vibrations in the first Y(0)-Y(1) shell (Fig. 7). The resulting potential includes the combination of double- and single-well potentials, similar to that in Ba_0.6_K_0.4_BiO_3_^52^. Moreover, the analysis has shown that the shape of the PRDF for 39 GPa at all the other temperatures, including 300 K, also differs from the Gaussian one, which is typical for harmonic oscillations. Therefore, we used the strong anharmonicity approximation in the treatment of the EXAFS spectrum, measured at room temperature, and to determine the shape and parameters of the vibration potential, as shown in Supplementary Note 6.

**Fig. 8:**
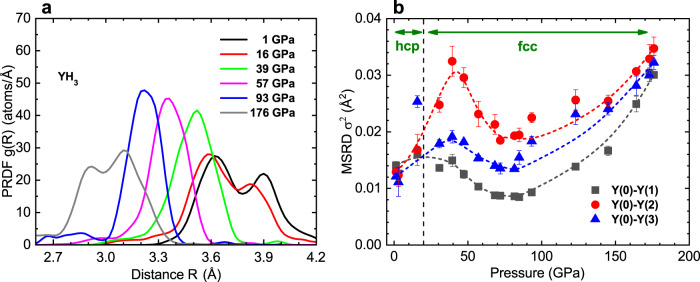
Pressure dependences of PRDF and MSRD. **a** The pair radial distribution function (PRDF) of the first Y(0)-Y(1) shell at different pressures up to 176 GPa; **b** The MSRD pressure dependence for the three nearest Y-Y shells around Y(0) atoms obtained for YH_3_ samples S1 and S2.

The PRDFs of the first Y(0)-Y(1) shell in the pressure range from 1 to 176 GPa, obtained for samples S1 and S2, are presented in Fig. 8a. The PRDF peak is split in the hcp phase (curves for 1 GPa and 16 GPa), while the splitting is absent in the fcc phase, in which the amplitude of the peak increases with an increase in pressure and the position shifts towards shorter distances. At the same time, the MSRD is growing for all three nearest Y-Y shells upon an increase in pressure from 20 to 40 GPa (Fig. 8b). This points to the local atomic rearrangement inside the fcc structure, which may be related to the local *C*2/*m*- type distortions in this pressure range.

The pressure increase in the range from 40 to 80 GPa is characterized by a monotonous decrease in the MSRD of all three nearest Y-Y shells around the absorbing atom Y(0), and a simultaneous increase in the amplitude and a decrease in the width of the PRDF of the first Y(0)-Y(1) shell (Fig. 8a, b). It is accompanied by the cardinal changes in the shape of the atomic vibrational potential. At ~80 GPa, two wells in the double-well potential merge to a single one, and yttrium atoms vibrate in the well-defined single-well (harmonic) potential shown in Supplementary Note 6. Thus, in the range 80–100 GPa, we observe the emergence of the most symmetric and ordered $Fm\bar{3}m$ (225) structure in the fcc phase of YH_3_. This result is strongly supported by the data of Raman spectroscopy, indicating the complete disappearance of all Raman active modes for both the vibrations of hydrogen and yttrium atoms (see Supplementary Note 3). A further increase in the pressure from 110 to 176 GPa causes a gradual decrease in the PRDF amplitude of the first Y(0)-Y(1) shell along with a shift of its peak towards shorter distances (Fig. 8a), while the MSRD increases for all three nearest Y-Y shells (Fig. 8b). At very high pressures (176 GPa), the PRDF maximum again splits into two peaks, which we attribute to the recurrence of the double-well potential for yttrium atom vibrations, as presented in Supplementary Note 6.

We would like to emphasize that the MSRD of the second nearest neighbor Y-Y shell significantly exceeds the value not only of the first, but also of the third ones (Fig. 8b). As can be seen from Fig. 2b, the second nearest Y-Y shell, with radius equal to the lattice parameter *a*, includes Y(0)-Y(2) and Y(1)-Y(1) interatomic bonds, which strongly differ from Y(0)-Y(1) and Y(0)-Y(3) ones because of the presence of the bonding hydrogen atom H_*o*_. As a rule^44,45^, in the EXAFS studies, the MSRD monotonously increases with as the interatomic distance increases. Therefore, the observed anomaly is an additional evidence of the effect of the hydrogen sublattice on the local structure of the yttrium one.

## Discussion

The multiscale length analysis of XAFS (short range), Raman (medium range), and XRD (long range) data allowed us to get insight into the nature of the phase transformations of the YH_3_ structure in the pressure range up to 180 GPa, concomitantly with the local electronic and atomic structure rearrangements.

Given that YH_3_ was predicted to be a superconductor with rather high *T*_*c*_ = 40 K at record low pressures of ~17 GPa in the fcc phase^16^, it could be placed into the same series together with other yttrium hydrides, which exhibit superconductivity at much higher pressures: *T*_*c*_ ~ 90 K in the YH_4_ phase at 120 GPa^55^, 227 K in YH_6_ and 243 K in YH_9_ at pressures above 200 GPa^3^. However, in YH_3_, the relatively strong electron-phonon coupling (*λ* ≈ 1.5^16^), which is a prerequisite for superconductivity, results in crystal lattice destabilization due to the Jahn-Teller (J-T) effect preventing superconductivity, as we discuss below. The possibility of such a scenario was mentioned previously for other materials^56,57^.

The pressure-dependent Y *K*-edge XANES (Fig. 3a,b) indicates that the compression of the YH_3_ lattice is accompanied by a reduction of the effective charge of yttrium ions and consequently leads to the insulator-to-metal transition. This behavior of YH_3_ is attributed to the pressure-induced formation of the Y(4*d*^1^)H_*o*_ complexes. The 4*d*^1^(Y^2+^) cations, which are formed above 20 GPa according to our XANES spectra, are J-T ‘active’^58,59^. Then it is the J-T effect that may have led to the absence of superconductivity in YH_3_, opening up a pseudo-gap in the DOS at the Fermi level due to the local distortions of *C*2/*m*- type at the hcp → fcc structural phase transition.

In more general terms, wondering why the seemingly promising MeH_*n*_ hydrides do not exhibit superconductivity, one should account for the charge balance between the metal and hydrogen atoms as well as the disorder and phase purity^60^. In particular, recent ab initio calculations indicate that when hydrogen atoms approach heavy metal atoms in hydrides under compression, the changes in the charge states arise for both metal and hydrogen atoms^61^. For example, it was reported that in LaH_10_ the electronic states near E_*F*_ exhibit a strong hybridization of the La 4*f* and H *s* orbitals, giving rise to a peculiar electrical charge characteristic of anionic La and both anionic H_1_ and cationic H_2_ atoms^61^.

EXAFS analysis has shown how the rearrangement of the nearest local environment of yttrium occurs with increasing pressure in the hcp → fcc phase transition region. Two yttrium positions in the hcp phase gradually merge into one, which is typical for the fcc phase, passing via the locally distorted nanostructure of the *C*2/*m*- type while XRD data on the long rage scale demonstrate the simple fcc structure. This manifests itself in the form of an increase in the MSRD of the three nearest yttrium coordination shells around the absorber atom (Fig. 8b), reaching maximum values at pressures near ~40 GPa. This corresponds to the completion of the hcp → fcc transition.

We have revealed that thermal vibrations of Y atoms in YH_3_ are strongly anharmonic at all pressures, except for the range 80–100 GPa. We consider the Jahn-Teller effect of 4*d*^1^(Y^2+^) (J − T) ions to be the origin of an anharmonicity because the Jahn-Teller ‘active’ 4*d*^1^(Y^2+^) ions are dynamically unstable, which creates distortion in the cubic lattice^58,59^.

The increase in pressure from 40 to 80 GPa leads to the transformation of 4*d*^1^(Y^2+^) ions to the stable ones 4*d*^2^(Y^1+^) and the double-well potential disappears (see Supplementary Note 6). Thus, we observe the most ordered fcc phase at a pressure range 80–100 GPa. The double-well potential appears again above 110 GPa (Supplementary Note 6), which is probably due to the appearance of the next 4*d*^3^(Y^0^) unstable Jahn-Teller ions.

In the pressure range 110–176 GPa, our XRD data show the appearance of partly unidentified XRD peaks (Supplementary Note 2). However, taking into account the XANES data, indicating the conservation of the fcc phase up to 176 GPa (Fig. 3), and the recurrence of a double-well potential of yttrium oscillations from the EXAFS spectra (Supplementary Note 6), we believe that the high-pressure structure should be associated with a dynamically distorted fcc phase. Moreover, we observed the appearance of sharp Raman hydrogen and deuterium modes in YH_3_ and YD_3_, respectively, with the predicted ratio 1.4 of frequencies at high pressure (Fig. 4c) while the samples were metallic (resistivity measurements data in Fig. 4a). This is an intriguing phenomenon and requires further study. Therefore, it cannot be ruled out that we have observed the starting process of the phase separation with the appearance of a small amount of nonmetallic Raman active phase (e.g. of *C*2/*m*- type) in the total metallic sample volume with the fcc structure.

Thanks to the combined XANES and EXAFS studies, we elucidated the nature of the hcp → fcc phase transition arising through Peierles-like local distortions in the range of 20–40 GPa, which is manifested by the appearance of the broad Raman lines that are forbidden in the perfect fcc structure of YH_3_ but allowed in the theoretically predicted disordered structure *C*2/*m*^43^. In the pressure range 0–20 GPa, the phase hcp → fcc transformation of YH_3_ structure detected by XRD on the long range scale is not completed. At ~20 GPa, the bandgap closes, but local distortions of the Peierls type give rise to additional scattering centers, due to which the resistance does not drop abruptly, but decreases gradually reaching minimum value at ~100–120 GPa instead. At both the medium range (Raman data) and short range (EXAFS data) scale, the transition *C*2/*m* → fcc stretched up to ~50 GPa accompanied with a gradual insulator-metal transition.

XANES analysis points to the pressure effect on the local electronic structure of YH_3_ via an increase in the density of yttrium *d* − *p* hybridized states accompanied by a change of formal yttrium valence from Y^3+^ to Y^2+^, which is due to effect of H^−^ anions and appearance of the dynamically unstable Jahn-Teller ‘active’ 4*d*^1^(Y^2+^) ions. This allowed us to explain the charge balance between the metal and hydrogen subsystems^60,61^ and the strong anharmonicity that manifests itself as vibrations of yttrium atoms in a double-well potential in our EXAFS analysis.

Anharmonicity is an important issue for understanding the mechanisms determining superconductivity in hydrides^10,62–65^. However, until now it has only been studied theoretically. For example, in SH_3_, considered to be a strongly anharmonic superconductor by Errea et al.^62^, the anharmonicity and quantum effects lead to a high symmetry structure^63^ and to a 30% decrease in the electron–phonon coupling^62^. Besides, as it was pointed by Struzhkin et al.^64^, the difference between experimental and calculated *T*_*c*_ values in YH_6_ may be due to strong anharmonicity and quantum effects, as recently discussed for LaH_10_ in Errea et al.^65^. In all these cases, the influence of strong anharmonicity was considered and attributed mainly to the hydrogen vibration. We suggest that not only hydrogen but also rare-earth ions vibrate in strongly anharmonic potential, as we experimentally showed above in YH_3_ from EXAFS.

In summary, we have demonstrated high efficiency of multiscale length analysis (XANES, EXAFS, XRD, and Raman scattering) complemented by resistivity measurements for the complex studies of metal hydrides under high pressures using archetype metal hydride YH_3_ as an example.

Taking into account the progress in the synthesis of LaH_10_, YH_6_, YH_9_ in nanodiamond anvils using pulsed laser heating and an increase in the X-ray intensity of the ESRF by an order of magnitude after the 2020 upgrade, in the next stage we plan to study the La *L*_3_-edge XANES spectra of LaH_10_ and the Y *K*-edge XANES and EXAFS spectra of YH_6_ and YH_9_. This will make it possible to establish a deeper understanding of the connection between the local peculiarities of the electronic and crystal structures, and the mechanism of high-temperature superconductivity in superhydrides.

## Methods

### Sample preparation and characterization

The YH_3_ powder samples were synthesized from 99.9% purity metallic yttrium by saturation with hydrogen under pressure about 100 bar and temperature 200 ^∘^C. The stoichiometry was YH_2.92(5)_, as measured by the gravimetric method. The pressure was generated using the diamond anvil cells equipped with nano-polycrystalline diamonds (NPDs)^40,41^. Two pressure runs, named samples S1 and S2, in which the single-bevel anvils with the culet size of 60 and 150 μm, respectively, were undertaken. In both runs, the gasket was made from MgO powder mixed with epoxy, which was confined into the steel T301 holder and preindented down to 15 μm thickness. The YH_3_ sample about 1/3 of the culet in size was placed into the gasket hole with no pressure medium.

Given that the samples S1 and S2 were of different effective thickness, their absorption coefficient *μ* had different jump values at the *K*-edge of yttrium (see Supplementary Note 1). The jump value of 0.5 for sample S2 turned out to be almost two times greater than for sample S1 (0.3), so most measurements under pressures up to 93 GPa were carried out on S2 run. With a further increase in pressure and cooling down to 10 K the NPDs broke. Therefore, measurements at higher pressures up to 176 GPa were carried out on sample S1. It should be noted here that the results obtained on both samples in the pressure range 0–93 GPa agree well.

The pressure was evaluated from the known equation of state (EOS) for YH_3_(Supplementary Note 2). The lattice parameters of the sample were determined from X-ray diffraction (XRD) data collected at 20 keV (*λ* = 0.62 Å) before each XAFS spectrum measurement. For sample S2, we performed the temperature-dependent XAFS measurements in the temperature range 10–300 K. Pressure was fixed as 39 GPa at room temperature and was not measured at low temperatures because of the small angle aperture of the cryostat. Based on our previous experience with this DAC design, we cannot rule out that at low temperature the pressure increases by 5–10 GPa. Nevertheless, at room temperature, both before and after the temperature run, the pressure was exactly the same: 39 GPa. Our XRD data, Raman, and resistivity measurement results are presented in Fig. 1a, b and Supplementary Notes 2 and 3.

### XAFS technique

X-ray absorption spectra above the Y *K*-edge (17038 eV) were collected at BM-23 microXAS beamline of European Synchrotron Radiation Facility (ESRF, Grenoble, France)^66^ in transmission mode. The double-crystal Si (111) monochromator was used for measurements, which allowed us to record EXAFS spectra with a very high signal-to-noise ratio of up to 16.5 Å^−1^ in momentum space. The energy step was 0.5 eV for XANES and 2 eV for EXAFS measurements. For low-temperature XAFS studies, the nano-diamond anvil cell was mounted in a liquid helium continuous flow cryostat with temperature control of ±1 K. To achieve maximum intensity and for higher harmonic rejection, a Pt coated mirror system in Kirck-Patrick-Baez geometry inclined to 3.5 mrad was used to focus the synchrotron radiation into a spot with a size of 5.7 μm × 7.4 μm FWHM, which was less than the size of the sample. Processing of the EXAFS spectra was carried using the ATHENA code^67^. Structural parameters were extracted by the Reverse Monte-Carlo (RMC) technique^45,46^ and in the case of strong anharmonicity we used the VIPER program package^54^. A brief description of these methods for the EXAFS spectra treatment is given in the Supplementary Notes 5 and 6. The XANES simulations for yttrium foil were performed within the full-multiple scattering formalism by the ab initio real-space FDMNES code^68,69^, and the results are presented in the Supplementary Note 4.

## Supplementary information

Supplementary Information

## Data Availability

All data supporting this study and its findings are available within the article and its Supplementary Information or from the corresponding authors upon reasonable request.

## Code availability

The code associated with this paper is available from the corresponding authors upon reasonable request.

## References

[1] Drozdov AP (2019). Superconductivity at 250 K in lanthanum hydride under high pressures. Nature.

[2] Somayazulu M (2019). Evidence for superconductivity above 260 K in lanthanum superhydride at megabar pressures. Phys. Rev. Lett..

[3] Kong, P.P. et al. Superconductivity up to 243 K in yttrium hydrides under high pressure. Preprint at http://arXiv.org/abs/1909.10482 (2019).

[4] Troyan, I.A. et al. Anomalous high-temperature superconductivity in YH_6_. Preprint at http://arXiv.org/abs/1908.01534v2 (2019). 10.1002/adma.202006832 33751670

[5] Drozdov AP, Eremets MI, Troyan IA, Ksenofontov V, Shylin SI (2015). Conventional superconductivity at 203 kelvin at high pressures in the sulfur hydride system. Nature.

[6] Liu LL, Sun HJ, Wang CZ, Lu WC (2017). High-pressure structures of yttrium hydrides. J. Phys.: Condens. Matter.

[7] Wang H, Li X, Gao G, Li Y, Ma Y (2018). Hydrogen-rich superconductors at high pressures. WIREs Comput. Mol. Sci..

[8] Snider E (2020). Room-temperature superconductivity in a carbonaceous sulfur hydride. Nature.

[9] Lv J, Sun Y, Liu H, Ma Y (2020). Theory-orientated discovery of high-temperature superconductors in superhydrides stabilized under high pressure. Matter Radiat. Extremes.

[10] Flores-Livas JA (2020). A perspective on conventional high-temperature superconductors at high pressure: methods and materials. Phys. Rep..

[11] Ashcroft NW (2004). Hydrogen dominant metallic alloys: high temperature superconductors?. Phys. Rev. Lett..

[12] Peng F (2017). Hydrogen clathrate structures in rare earth hydrides at high pressures: possible route to room-temperature superconductivity. Phys. Rev. Lett..

[13] Huiberts J (1996). Yttrium and lantanium hydride films with switchable optical properties. Nature.

[14] Machida A (2006). X-ray diffraction investigation of the hexagonal-fcc structural transition in yttrium trihydride under hydrostatic pressure. Solid State Commun..

[15] Jarosik MW, Szcześniak R, Wrona IA, Kostrzewa M (2017). Non-BCS superconducting state in yttrium hydride at a record low value of the external pressure. Solid State Commun..

[16] Kim DY, Scheicher RH, Ahuja R (2009). Predicted high-temperature superconducting state in the hydrogen-dense transition-metal hydride YH_3_ at 40 K and 17.7 GPa. Phys. Rev. Lett..

[17] Matsuoka T (2007). Electrical properties of YH_3_ under high pressure. J. Phys. Soc. Jpn.

[18] Ohmura A (2006). Infrared spectroscopic study of the band-gap closure in YH_3_ at high pressure. Phys. Rev. B.

[19] Palasyuk T, Tkacz M (2005). Hexagonal to cubic phase transition in YH_3_ under high pressure. Solid State Commun..

[20] Machida A, Ohmura A, Watanuki T, Aoki K, Takemura K (2007). Long-period stacking structures in yttrium trihydride at high pressure. Phys. Rev. B.

[21] Kume T (2007). High-pressure study of YH_3_ by Raman and visible absorption spectroscopy. Phys. Rev. B.

[22] Kume T (2011). High-pressure study of ScH_3_: Raman, infrared, and visible absorption spectroscopy. Phys. Rev. B.

[23] Kierey H, Rode M, Jacob A, Borgschulte A, Schoenes J (2001). Raman effect and structure of YH_3_ and YD_3_ thin epitaxial films. Phys. Rev. B.

[24] Udovic T, Huang Q, Rush J (1996). Characterization of the structure of YD_3_ by neutron powder diffraction. J. Phys. Chem. Solids.

[25] Fedotov VK, Antonov VE, Bashkin IO, Hansen T, Natkaniec I (2006). Displacive ordering in the hydrogen sublattice of yttrium trihydride. J. Phys.: Condens. Matter.

[26] Udovic TJ, Huang Q, Santoro A, Rush JJ (2008). The nature of deuterium arrangements in YD_3_ and other rare-earth trideuterides. Z. Kristallogr. Cryst. Mater..

[27] Lengeler B (1984). Lattice site location of hydrogen by use of extended X-ray absorption fine structure. Phys. Rev. Lett..

[28] McCaulley JA (1993). In-situ x-ray absorption spectroscopy studies of hydride and carbide formation in supported palladium catalysts. J. Phys. Chem..

[29] Tew MW, Miller JT, van Bokhoven JA (2009). Particle size effect of hydride formation and surface hydrogen adsorption of nanosized palladium catalysts: L_3_ edge vs K edge X-ray absorption spectroscopy. J. Phys. Chem. C.

[30] Ngene P, Longo A, Mooij L, Bras W, Dam B (2017). Metal-hydrogen systems with an exceptionally large and tunable thermodynamic destabilization. Nat. Commun..

[31] Davoli I (1989). Palladium L_3_ absorption edge of PdH_0.6_ films: Evidence for hydrogen induced unoccupied states. Solid State Commun..

[32] Ishimatsu N (2009). Effect of hydrogenation on the electronic state of metallic La hydrides probed by X-ray absorption spectroscopy at the La L-edges.. J. Phys.: Conf. Ser..

[33] Fieser ME (2017). Evaluating the electronic structure of formal Ln^*I**I*^ ions in Ln^*I**I*^(C_5_H_4_SiMe_3_)$${\,}_{3}^{1-}$$ using XANES spectroscopy and DFT calculations. Chem. Sci..

[34] Matsumoto K (2011). Noncollinear spin structure in Fe–Ni invar alloy probed by magnetic EXAFS at high pressure. J. Phys. Soc. Jpn.

[35] Baldini M (2011). High-pressure EXAFS measurements of crystalline Ge using nanocrystalline diamond anvils. Phys. Rev. B.

[36] Properzi L, Cicco AD, Nataf L, Baudelet F, Irifune T (2015). Short-range order of compressed amorphous GeSe_2_. Sci. Rep..

[37] Fabbris G, Hücker M, Gu GD, Tranquada JM, Haskel D (2016). Combined single crystal polarized XAFS and XRD at high pressure: probing the interplay between lattice distortions and electronic order at multiple length scales in high-T_*c*_ cuprates. High Pressure Res..

[38] Rosa AD (2018). Effect of the fcc-hcp martensitic transition on the equation of state of solid krypton up to 140 GPa. Phys. Rev. B.

[39] Kuramochi K, Ishimatsu N, Sakai T, Kawamura N, Irifune T (2020). An application of NPD to double-stage diamond anvil cells: XAS spectra of rhenium metal under high pressures above 300 GPa. High Pressure Res..

[40] Irifune T, Kurio A, Sakamoto S, Inoue T, Sumiya H (2003). Ultrahard polycrystalline diamond from graphite. Nature.

[41] Ishimatsu N (2016). Applications of nano-polycrystalline diamond anvils to X-ray absorption spectroscopy under high pressure. High Press. Res..

[42] Crane MJ (2019). High-pressure, high-temperature molecular doping of nanodiamond. Sci. Adv..

[43] Yao Y, Klug DD (2010). Consecutive Peierls distortions and high-pressure phase transitions in YH_3_. Phys. Rev. B.

[44] Cicco AD, Filipponi A, Itié JP, Polian A (1996). High-pressure EXAFS measurements of solid and liquid Kr. Phys. Rev. B.

[45] Timoshenko J (2018). Neural network approach for characterizing structural transformations by X-ray absorption fine structure spectroscopy. Phys. Rev. Lett..

[46] Timoshenko J, Kuzmin A, Purans J (2014). EXAFS study of hydrogen intercalation into ReO_3_ using the evolutionary algorithm. J. Phys.: Condens. Matter.

[47] Sevillano E, Meuth H, Rehr JJ (1979). Extended x-ray absorption fine structure Debye-Waller factors. I. Monatomic crystals. Phys. Rev. B.

[48] Mustre de Leon J, Conradson SD, Batistić I, Bishop AR (1990). Evidence for an axial oxygen-centered lattice fluctuation associated with the superconducting transition in YBa_2_Cu_3_O_7_. Phys. Rev. Lett..

[49] Bishop AR, Mihailovic D, de Leon JM (2003). Signatures of mesoscopic Jahn-Teller polaron inhomogeneities in high-temperature superconductors. J. Phys.: Condens. Matter.

[50] Menushenkov A (2010). Correlation of the local and the macroscopic properties of high-temperature superconductors. Z. Kristallogr. Cryst. Mater..

[51] Menushenkov AP, Klement’ev KV, Konarev PV, Meshkov AA (1998). Anharmonicity and superconductivity in Ba_0.6_K_0.4_BiO_3_. JETP Lett..

[52] Menushenkov AP, Klementev KV (2000). Extended x-ray absorption fine-structure indication of a double-well potential for oxygen vibration in Ba_1−*x*_K_*x*_BiO_3_. J. Phys.: Condens. Matter.

[53] Ivanov VG, Ivanov AA, Menushenkov AP, Joseph B, Bianconi A (2016). Fe–As bond fluctuations in a double-well potential in LaFeAsO. J. Supercond..

[54] Klementev KV (2001). Extraction of the fine structure from x–ray absorption spectra. J. Phys. D Appl. Phys..

[55] Li Y (2015). Pressure-stabilized superconductive yttrium hydrides. Sci. Rep..

[56] Moussa JE, Cohen ML (2006). Two bounds on the maximum phonon-mediated superconducting transition temperature. Phys. Rev. B.

[57] Besedin SP, Jephcoat AP, Irodova AV (2011). Light-induced phase transition in AlD_3_ at high pressure. Phys. Rev. B.

[58] Goodenough JB (1998). Jahn–Teller phenomena in solids. Annu. Rev. Mater. Sci..

[59] Wolf W, Herzig P (2000). First-principles investigations of transition metal dihydrides, TH_2_: T = Sc, Ti, V, Y, Zr, Nb; energetics and chemical bonding. J. Phys.: Condens. Matter.

[60] Harshman DR, Fiory AT (2020). High-*T*_*c*_ superconductivity in hydrogen clathrates mediated by coulomb interactions between hydrogen and central-atom electrons. J. Supercond. Novel Magn..

[61] Liu L (2019). Microscopic mechanism of room-temperature superconductivity in compressed LaH_10_. Phys. Rev. B.

[62] Errea I (2015). High-pressure hydrogen sulfide from first principles: a strongly anharmonic phonon-mediated superconductor. Phys. Rev. Lett..

[63] Errea I (2016). Quantum hydrogen-bond symmetrization in the superconducting hydrogen sulfide system. Nature.

[64] Struzhkin V (2020). Superconductivity in La and Y hydrides: remaining questions to experiment and theory. Matter Radiat. Extremes.

[65] Errea I (2020). Quantum crystal structure in the 250-kelvin superconducting lanthanum hydride. Nature.

[66] Mathon O (2015). The time-resolved and extreme conditions XAS (TEXAS) facility at the European Synchrotron Radiation Facility: the general-purpose EXAFS bending-magnet beamline BM23. J. Synchrotron Radiat..

[67] Ravel B, Newville M (2005). *ATHENA*, *ARTEMIS*, *HEPHAESTUS*: data analysis for X-ray absorption spectroscopy using *IFEFFIT*. J. Synchrotron Radiat..

[68] Joly Y (2001). X-ray absorption near-edge structure calculations beyond the muffin-tin approximation. Phys. Rev. B.

[69] Bunău O, Joly Y (2009). Self-consistent aspects of x-ray absorption calculations. J. Phys.: Condens. Matter.

