# Supplementary material for: Local electronic structure rearrangements and strong anharmonicity in YH3 under pressures up to 180 GPa
Source: Nat Commun. 2021 Mar 19;12:1765. doi: 10.1038/s41467-021-21991-x (PMC7979761; doi:10.1038/s41467-021-21991-x)
Supplement: Supplementary file 1 — Supplementary Information [file 41467_2021_21991_MOESM1_ESM.pdf]

# Supplementary information
# for
# Local electronic structure rearrangements and strong anharmonicity in $YH_3$ under pressures up to 180 GPa

J. Purans, A.P. Menushenkov, S. Besedin, A.A. Ivanov, V. Minkov,
I. Pudza, A. Kuzmin, K.V. Klementiev, S. Pascarelli,
O. Mathon, A. D. Rosa, T. Irifune, M.I. Eremets

## Supplementary Note 1.
## XAFS technique, Initial XAS spectra, Normalized XANES Spectra

XAFS represents a fine oscillating structure in the spectral dependence of the X-ray absorption coefficient, arising as a result of interference of the outgoing and reflected photoelectron waves around absorbing atom. XAFS includes two regions: X-ray absorption near edge structure – XANES, arising from multiple scattering of photoelectron on the nearest environment of absorbing atom, and extended X-ray absorption fine structure – EXAFS, arising mainly from single scattering processes. The XANES region provides information on the local electronic structure, the valence state of ions, and the lattice symmetry, while EXAFS gives the local crystal structure description including the radii, coordination numbers and EXAFS Debye-Waller factors $\exp(-\sigma^2 k^2)$, where $\sigma^2$ is the mean-square relative displacement (MSRD) of absorber and back-scatterer atoms of the nearest coordination shells around the absorbing atom. MSRD is the most adequate characteristic of both static and dynamic disorder of the structure. XAFS is a fast method with the probing time of about $10^{-15}$ s and is the most suitable for the dynamic effects study in comparison, for example, with Mössbauer spectroscopy,[S1] characterized by the probing time of about $10^{-9}$ s, which is much longer than the usual time scale of electronic dynamics in solids.

X-ray ansorption spectra (XAS) were measured above the Y $K$-absorption edge (17038.4 eV) using double crystal Si(111) monochromator with the energy step 0.5 eV at XANES and about 2-3 eV at EXAFS region with a 0.4 mm slit. The initial X-ray absorption spectra at the Y $K$-edge of $YH_3$ for samples S1 and S2 at different pressures are presented in Supplementary Fig.**S1**. The magnitudes of jumps for both samples did not change with increasing pressure, which indicates that the amount and the effective thickness of the both samples do not change during the measurements over the entire pressure range. It means that there is no extrusion of the samples from diamond anvils during the experiment.

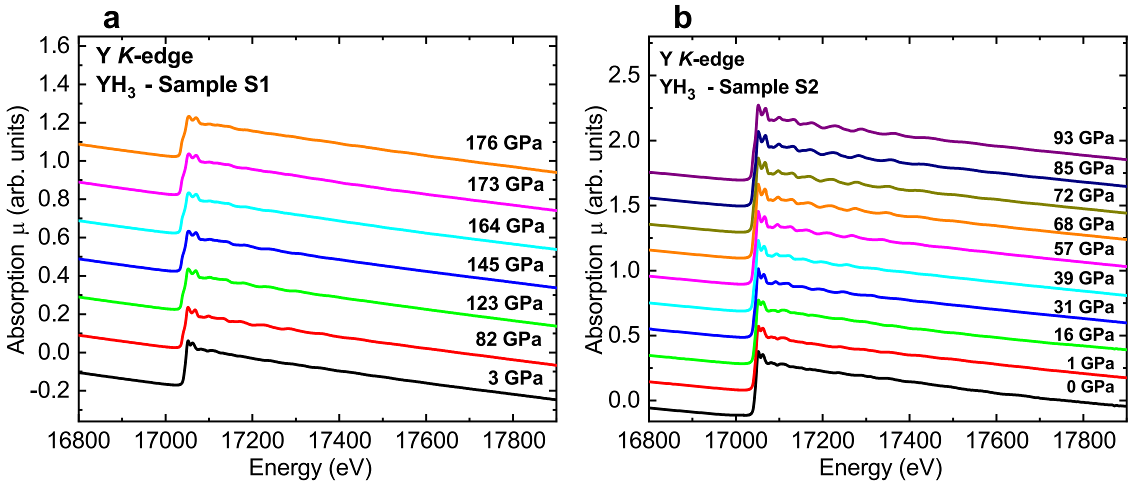


**Supplementary Figure S1**: **Initial XAS spectra.** The initial X-ray absorption spectra at the Y $K$-edge of $YH_3$ for samples S1 (**a**) and S2 (**b**) at different pressures.

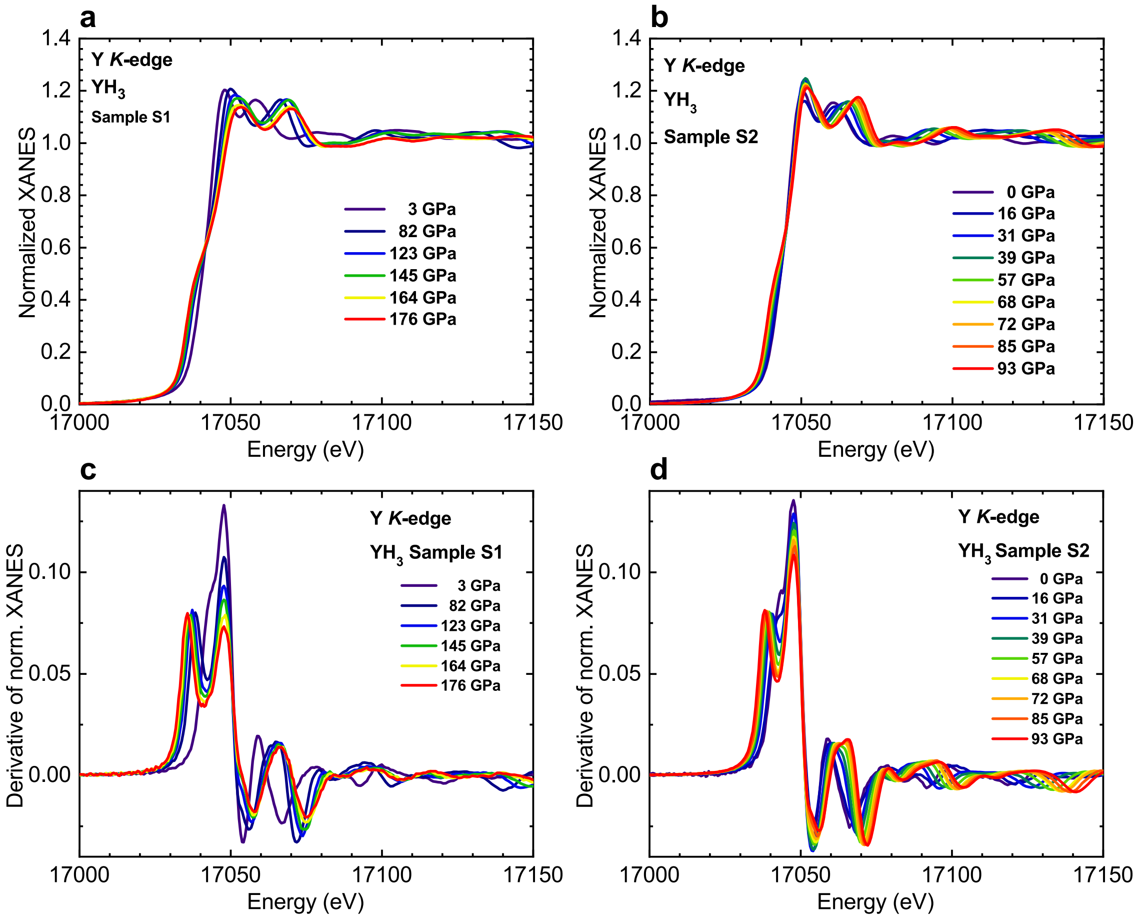


**Supplementary Figure S2**: **XANES spectra.** Pressure-dependent experimental Y *K*-edge XANES spectra (**a** and **b**) and their first derivatives (**c** and **d**) for $YH_3$ samples S1 and S2.

# Supplementary Note 2.
# XRD Data and pressure determination

For pressure determination, the lattice parameters of the sample were obtained from X-ray diffraction (XRD) measurements made concomitantly with collecting the XAS spectra. For the XRD measurements, the beam was switched to 20 keV ($\lambda = 0.62$ Å). The pressure was evaluated from the known pressure-volume (P-V) equation of state (EOS) for $YH_3$. For the hcp phase, the Birch-Murnagan EOS was used with the parameters: $V_0 = 23.39$ cm$^3$/mole (38.675 Å$^3$/f.u.), $K_0 = 71.9$ GPa, $K_0' = 5.0$[S2] . For the fcc phase the P-V data from a separate XRD study in the region 17 - 180 GPa (Supplementary Fig. **S4**) complemented with the ambient-pressure volume per formula unit $V_0 = 36.87$ Å$^3$/f.u. from Ref.[S3] were fit with the Vinet function with $V_0$ fixed.[S4] The obtained bulk modulus and its derivative respectively are: $K_0 = 91.17$ GPa, $K_0' = 3.576$.

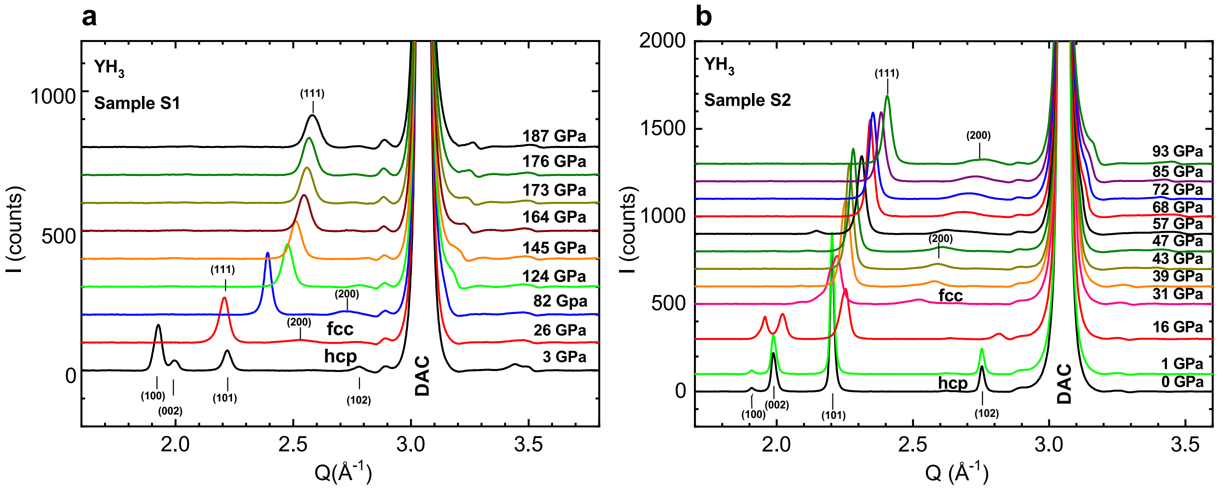


**Supplementary Figure S3**: **XRD data.** X-ray diffraction patterns for $YH_3$, samples S1 (**a**) and S2 (**b**).

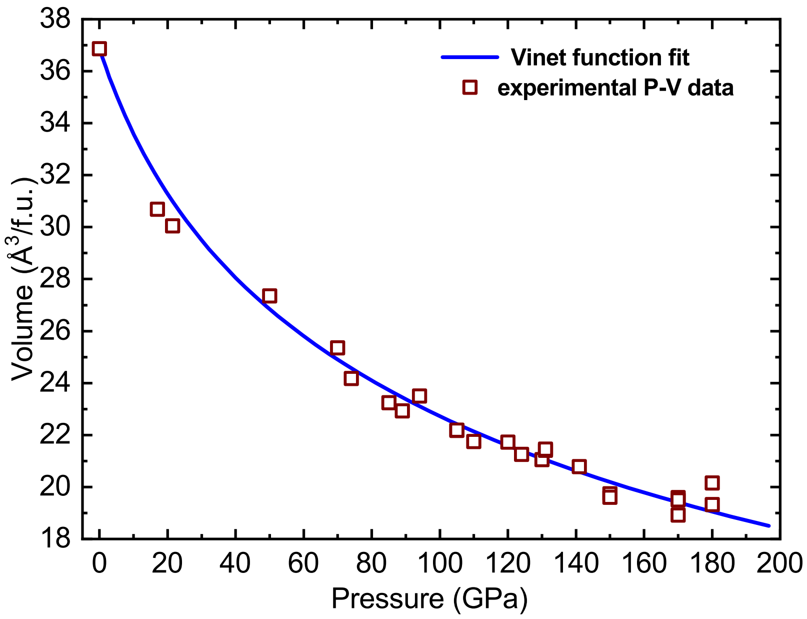


**Supplementary Figure S4**: **EOS.** Room temperature equation of state for fcc $YH_3$.

# Supplementary Note 3.
# Raman scattering

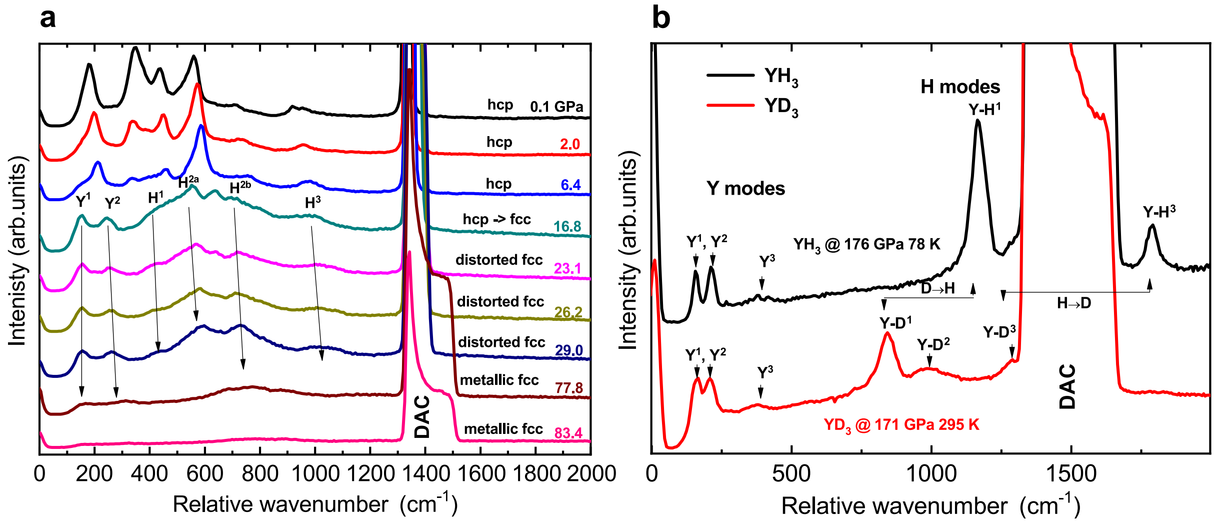


**Supplementary Figure S5**: **Raman scattering.** Raman scattering spectra of $YH_3$ and $YD_3$. **a** Pressure dependence (up to 83.4 GPa) of the Raman scattering in $YH_3$; **b** The Raman scattering in $YH_3$ at 176 GPa, 78 K and $YD_3$ at 171 GPa, 295 K.

# Supplementary Note 4.
# Y *K*-edge XANES calculations for metallic yttrium

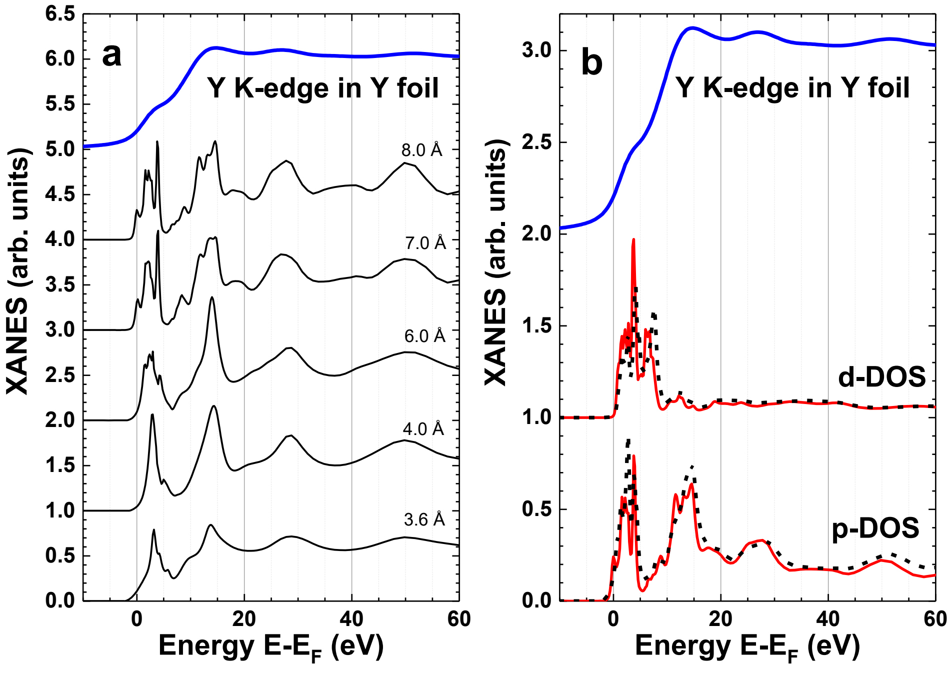


**Supplementary Figure S6**: **XANES calculations.** **a** Comparison of the experimental Y *K*-edge XANES for yttrium foil (upper curve) at 300 K with the results of the full-multiple scattering (FMS) calculations by the FDMNES code[S5,S6] as a function of the cluster radius R = 3.6, 4.0, 6.0, 7.0, 8.0 Å around the absorbing yttrium atom. **b** Comparison of the calculated partial $p$(Y) and $d$(Y) density of states (DOS) with the experimental Y *K*-edge XANES: solid curves – excited state DOS, dashed curves – ground state DOS. The energy scale is relative to the theoretical Fermi level $E_F$.

The Y *K*-edge XANES spectra of metallic yttrium (hcp phase) were calculated within the full-multiple scattering (FMS) formalism by the ab initio real-space FDMNES code.[S5,S6] The calculations were performed using real energy-dependent Hedin-Lundqvist exchange-correlation potential[S7] and the self-consistent cluster potential for the clusters with radii R = 3.6, 4.0, 6.0, 7.0, 8.0 Å around the absorbing yttrium atom. The results of the calculations are compared

with the experimental spectrum of yttrium foil measured at 300 K in Supplementary Fig. **S6**(a). To identify the origin of the XANES peaks, the partial $p$(Y) and $d$(Y) density of states (DOS) are shown in Supplementary Fig. **S6**(b) for the excited and ground states, i.e., with and without the core hole at the $1s$(Y) level of the absorbing yttrium atom.

As one can see, the core hole effect is relatively small. All main features of the experimental Y $K$-edge XANES of yttrium foil are reproduced by a small cluster of 3.6 Å radius containing six yttrium atoms of the first coordination shell only. The shoulder at the absorption edge is due to the mixed $p - d$ states of yttrium, whereas other peaks located above the edge are due to the features in $p$(Y)-DOS.

## Supplementary Note 5.
## Reverse Monte Carlo (RMC) analysis of EXAFS spectra

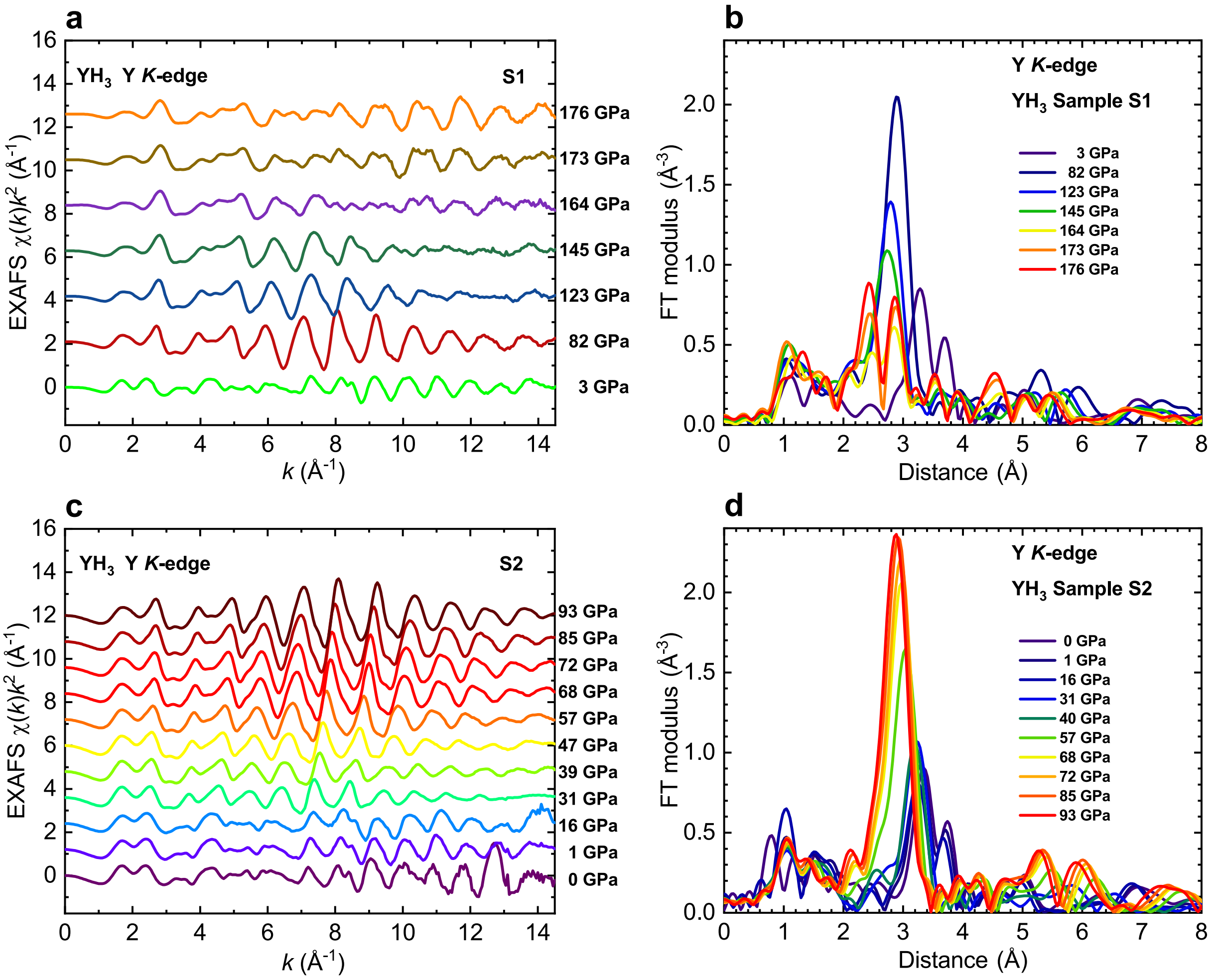


**Supplementary Figure S7**: **EXAFS spectra.** Pressure dependence of the experimental Y $K$-edge EXAFS spectra $\chi(k)k^2$ (**a** and **c**) and their Fourier transform moduli (**b** and **d**) for $YH_3$ samples S1 and S2. The FTs were not corrected for any phase shifts; therefore, the positions of peaks differ from crystallographic values.

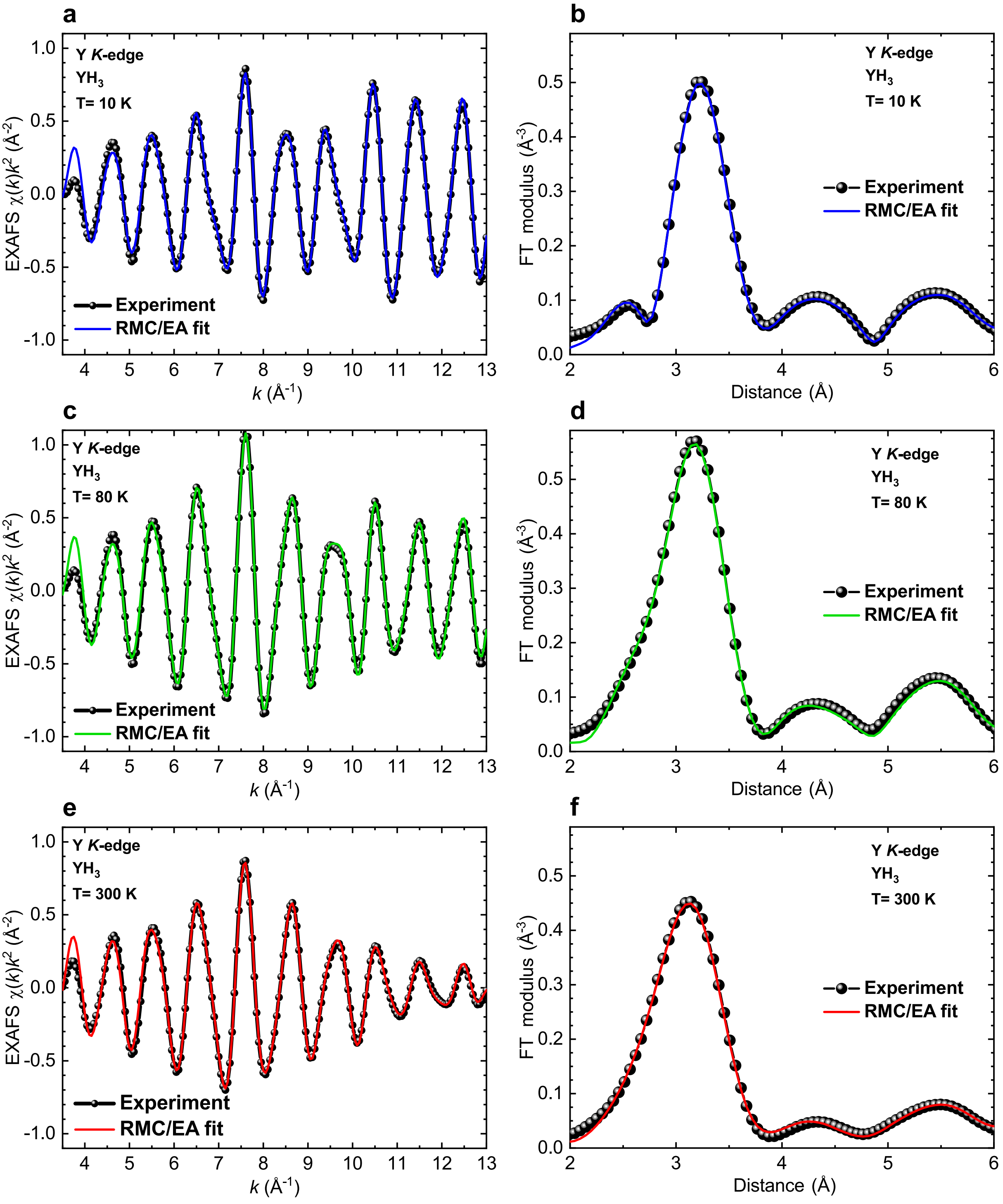


**Supplementary Figure S8**: **RMC EXAFS analysis.** The results of RMC/EA-EXAFS calculations for fcc-$YH_3$ at selected temperatures: circles - experimental data, lines - calculated spectra. The Y $K$-edge EXAFS spectra $\chi(k)k^2$ of $YH_3$ are compared in the left panels and their Fourier transform moduli - in the right panels.

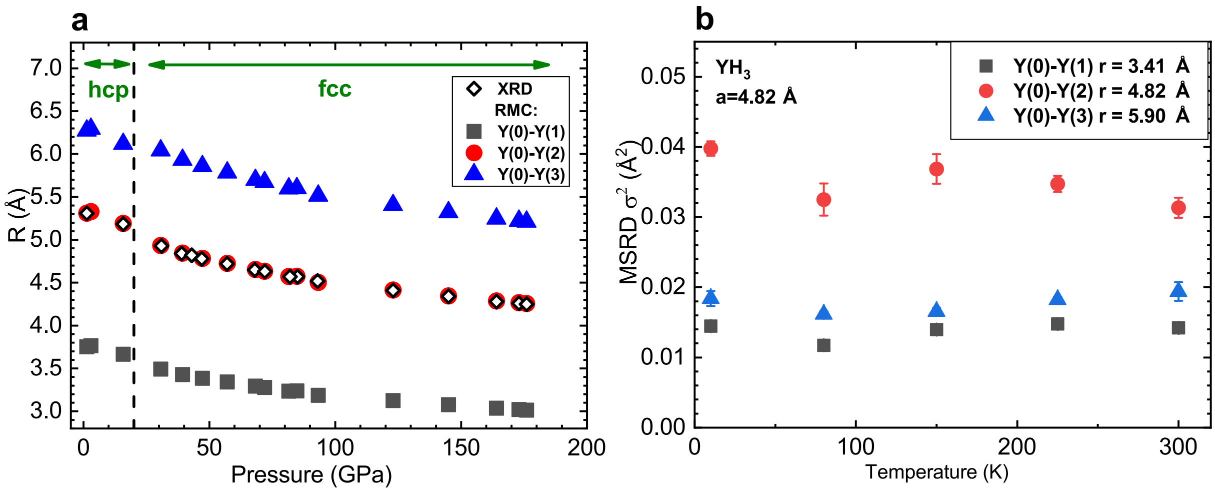


**Supplementary Figure S9**: **Structure characteristics.** **a** Pressure dependence of the three nearest Y–Y shells radii and **b** temperature dependence of MSRD at 39 GPa for three nearest Y–Y shells, obtained from the RMC analysis of the Y $K$-edge EXAFS spectra of $YH_3$.

The EXAFS oscillations were extracted using the ATHENA code,[S8] taking care to remove the low-frequency oscillations. The EXAFS-functions $\chi(k)k^2$ obtained from absorption spectra were Fourier transformed using Kaiser-Bessel windowing function in the range of wavenumber $k$ from 1.5 to 16.5 Å$^{-1}$.

## RMC simulations

The experimental Y $K$-edge EXAFS spectra were analysed using the RMC method with an evolutionary algorithm (EA) approach, as implemented in the EvAX code.[S9] In the RMC method, the material is represented by a 3D structural model, and atomic coordinates are randomly changed at each iteration of the simulation to minimize the difference between experimental and configuration-averaged calculated EXAFS spectra.

The initial models of $YH_3$ structure at different pressures were constructed based on the lattice parameters estimated from the experimental diffraction data. For instance, the model of the low-pressure hcp $YH_3$ phase was constructed based on the structure determined by neutron powder diffraction for the hcp $YD_3$ phase with the space group $P\bar{3}c1$ (163)[S10] leaving the Wyckoff positions of atoms but modifying the lattice parameters a and c. The model of the high-pressure fcc $YH_3$ phase was constructed by changing the lattice constant a and leaving the Wyckoff positions corresponding to the cubic $Fm\bar{3}m$ (225) phase.

The RMC simulations of pressure-dependent EXAFS data were performed with the $3 \times 3 \times 3$ large supercell containing 648 atoms for the hcp structure or 432 atoms for the fcc structure. Temperature-dependent EXAFS data measured at the pressure of 39 GPa were simulated for the fcc structure using the $4 \times 4 \times 4$ large supercell containing 1024 atoms. Periodic boundary conditions were applied to avoid surface related effects. 32 atomic configurations were used simultaneously in the EA method. At each iteration, a new atomic configuration was generated by randomly displacing all atoms in the supercell with a maximum permissible displacement of 0.4 Å.

The configuration-averaged Y $K$-edge EXAFS spectra were calculated by *ab initio* real-space FEFF8.50L code[S11] including multiple-scattering contributions up to the 4-th order. The complex energy-dependent exchange-correlation Hedin-Lundqvist potential[S7] was employed to account for inelastic effects. The amplitude reduction factor $S_0^2$ was set to 1.0. As a criterion for the agreement between the experimental and calculated EXAFS spectra, we used a comparison of their Morlet wavelet transforms.[S12] Calculations were performed in the $k$-space range from 3.5 to 13 Å$^{-1}$ and the R-space ranges from 2 to 6 Å for the fcc structure and from 2.5 to 7.5 Å for the hcp structure. No significant improvement in the agreement was observed after 3000 iterations. At least three RMC/EA simulations with different sequences of pseudo-random numbers were performed for each experimental data set.

The RMC simulations performed with and without hydrogen atoms gave close agreement with the experimental data, indicating that due to hydrogen is a weak scatterer, its contribution cannot be reliably evidenced. Therefore, further discussion will be limited to the Y–Y correlations. As a result of the RMC simulations, sets of atomic coordinates were obtained. They were used to calculate partial pair radial distribution functions (PRDFs) $g_{\text{Y-Y}}(\text{R})$ and to estimate structural parameters, as interatomic distances R(Y–Y) and mean-square relative displacement (MSRD) factors $\sigma^2$(Y–Y) for Y–Y atom pairs.

# Supplementary Note 6.
# EXAFS spectra analysis in the case of strong anharmonicity

The experimental Y $K$-edge EXAFS spectra treatments for the first Y(0)–Y(1) shell around the absorbing atom of $YH_3$ were analysed in real-space using VIPER program package.[S13] The EXAFS functions $\chi(k)k^2$ obtained from the absorption spectra were Fourier transformed in the range of wavenumber $k$ from 1.5 to 16.5 Å$^{-1}$, using the Kaiser–Bessel windowing function. Fourier back-transformation (BFT) was carried out using a Hanning window in the real-space range corresponding to the nearest interatomic Y(0)–Y(1) distance. The model EXAFS function was fitted to the BFT filtered experimental one.

The model EXAFS function $\chi(k)$ for atomic pair absorber–scatterer oscillations is constructed as follows. Suppose we know the potential of these oscillations as a parametric function of interatomic distance. Solving the stationary Schrödinger equation numerically for the particle with the reduced mass of the atomic pair, one obtains a pair radial distribution function (PRDF) of atoms in the $i$-th shell:

$$g_i(r) = N_i \sum_n |\Psi_n(r)|^2 e^{-E_n/kT} / \sum_n e^{-E_n/kT} \qquad (1)$$

where $N_i$ is the coordination number, and $E_n$ and $\Psi_n$ are the $n$-th energy level and its corresponding wave function. Given the PRDFs, the model EXAFS function is calculated as

$$\chi(k) = \frac{1}{k} \sum_i F_i(k) \int_{r_{\min}}^{r_{\max}} g_i(r) \sin[2kr + \phi_i(k)]/r^2 \, dr \qquad (2)$$

where $k = \sqrt{2m_e/\hbar^2(E - E_{th})}$ is the photoelectron wavenumber referenced to the ionization threshold $E_{th}$, and $r_{\min}$ and $r_{\max}$ are determined by the windowing function of the back Fourier transform. The phase shift $\phi_i(k)$ and the scattering amplitude $F_i(k)$ were calculated using the FEFF8.50L code[S11] for the fcc structure of $YH_3$, using the $4 \times 4 \times 4$ large supercell containing 1024 atoms with crystallographic data from neutron diffraction study[S10] and our s-XRD data. The potential parameters were extracted from the model-to-experimental EXAFS-function fits.

The model for the oscillatory Y-Y potential for the $YH_3$ *C/2m* phase at 39 GPa was constructed as follows. Let us imagine the position of the angular Y(0) atoms as fixed, and the octahedron of 6 Y(1) atoms in the centers of the faces (see **Fig. 5b** in the main text), squeezed to the center of the cube. In six neighboring cells being joined through the faces of the cube, these internal octahedra will, on the contrary, be stretched. This compression-tension occurs dynamically and, apparently, due to the dynamic instability of the hydrogen subsystem. Such a movement exchanges the roles of two inequivalent Y positions and requires a double-well form of the oscillatory Y-Y potential. Here, we take a parabolic form for each well, $U_1 = k_1(r - r_1)^2/2$ and $U_2 = k_1(r - r_2)^2/2$, and these are joined continuously.

Angular atoms Y(0), of which a fraction of 1/4, see this displacement perpendicular to the nearest neighbor. Therefore, they do not fill the double-well potential and oscillate in a single-well one. Atoms Y(1) in the octahedron, of which a fraction of 3/4, are seen by 4 atoms at a short distance, 4 at a long distance and 4 (almost at the face of a cube) on average distance. Total, coordination number with short distances $= 12 \times 3/4 \times 1/3 = 3$, the same as for long ones. For medium: $12 \times (1/4 + 3/4 \times 1/3) = 6$. I.e. as a result, we have one single-well potential with a coordination number of 6 and one double-well potential, also with the number 6 (3 in each well). Given the calculated $\chi(k)$, defined by equations (1) and (2) in addition to single-well potential, we performed a least-squares fit between the model and experimental $\chi(k)k^2$ over the range k = 2-16 Å$^{-1}$ and extracted the full potential parameters (see **Fig. 7** in the main text).

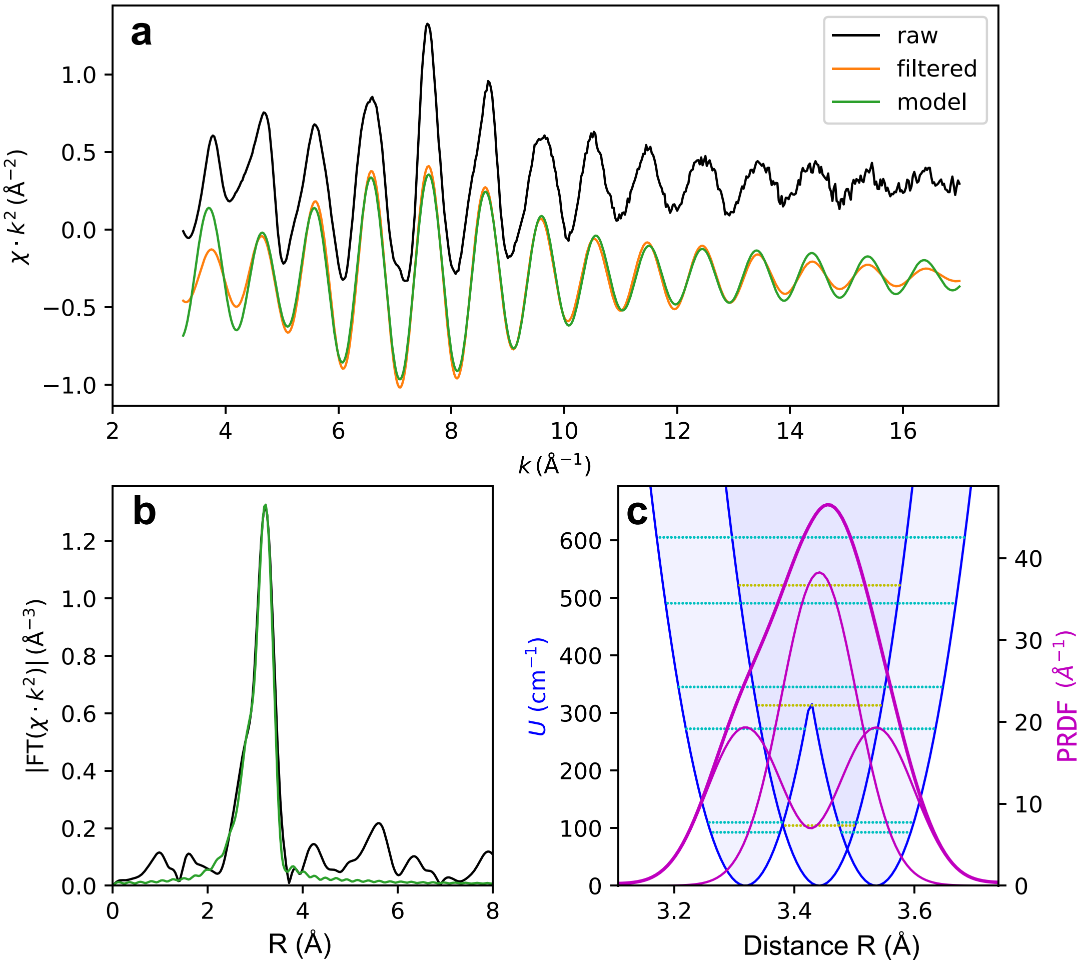


**Supplementary Figure S10**: **An anharmonic potential.** The results of Y *K*-edge EXAFS-spectra treatments for $YH_3$ at 39 GPa and 300 K.
**a** The raw experimental (black) and filtered (red) EXAFS-function $\chi(k)k^2$ after back Fourier transform (BFT) in the real-space range (2.2-3.8 Å) with the model (green);
**b** Fourier transform (FT) modulus of EXAFS-function $\chi(k)k^2$ (black) and the result of fitting (green) of the first Y(0)–Y(1) shell in the real-space range (2.2-3.8 Å);
**c** The double-well and single-well potentials (blue) of Y atom vibrations and their pair radial distribution functions (PRDF) (dark magenta) for the Y(0)–Y(1) bond in the first coordination shell. The resulting PRDF is shown by bold dark magenta line, while the energy levels are shown by yellow for single-well and by light blue for the double-well potential.

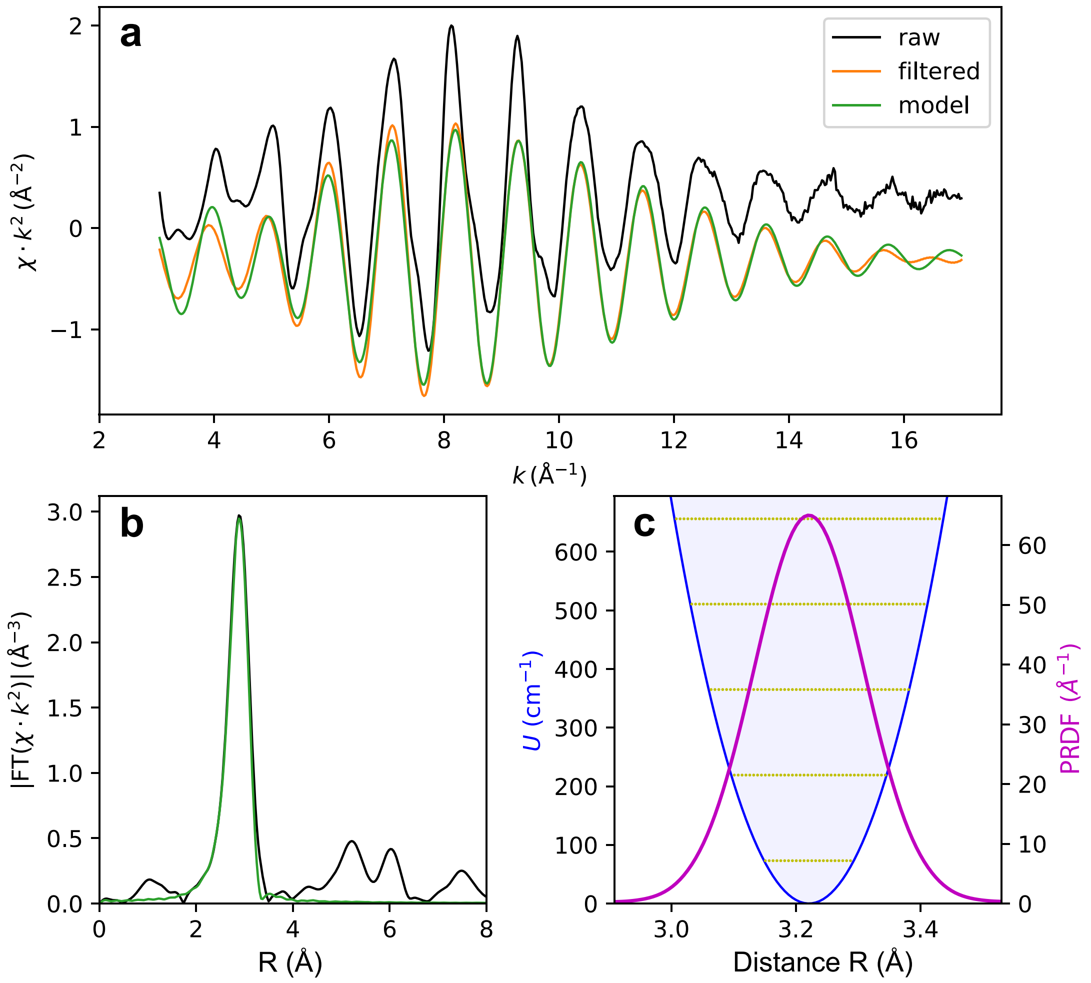


**Supplementary Figure S11**: **A harmonic potential.** The results of Y *K*-edge EXAFS-spectra treatments for $YH_3$ at 93 GPa and 300 K.
**a** The raw experimental (black) and filtered (red) EXAFS-function $\chi(k)k^2$ after back Fourier transform (BFT) in the real-space range (1.8-3.5 Å) with the model (green);
**b** Fourier transform (FT) modulus of EXAFS-function $\chi(k)k^2$ (black) and the result of fitting (green) of the first Y(0)–Y(1) shell in the real-space range (2.2-3.8 Å);
**c** The single-well potentials (blue) of Y atom vibrations and their pair radial distribution functions (PRDF) (dark magenta) for the Y(0)–Y(1) bond in the first coordination shell. The energy levels are shown by yellow.

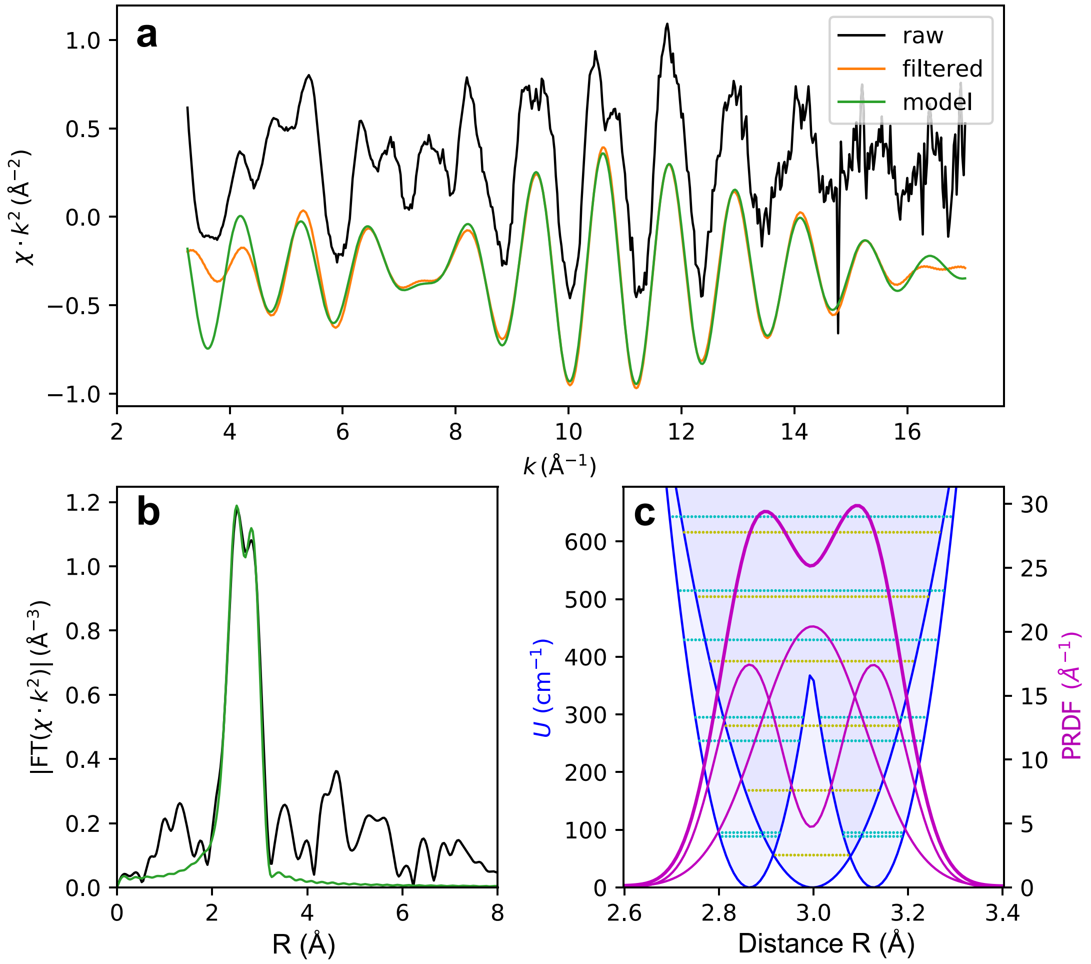


**Supplementary Figure S12**: **The recurrence of an anharmonic potential.** The results of Y $K$-edge EXAFS-spectra treatments for $YH_3$ at 176 GPa and 300 K.

**a** The raw experimental (black) and filtered (red) EXAFS-function $\chi(k)k^2$ after back Fourier transform (BFT) in the real-space range (1.8-3.2 Å) with the model (green);

**b** Fourier transform (FT) modulus of EXAFS-function $\chi(k)k^2$ (black) and the result of fitting (green) of the first Y(0)–Y(1) shell in the real-space range (2.2-3.8 Å);

**c** The double-well and single-well potentials (blue) of Y atom vibrations and their pair radial distribution functions (PRDF) (dark magenta) for the Y(0)–Y(1) bond in the first coordination shell. The resulting PRDF is shown by bold dark magenta line, while the energy levels are shown by yellow for single-well and by light blue for the double-well potential.

# Supplementary References


S1. Troyan, I. et al. Observation of superconductivity in hydrogen sulfide from nuclear resonant scattering. *Science* **351**, 1303–1306 (2016).

S2. Machida, A. et al. X-ray diffraction investigation of the hexagonal–fcc structural transition in yttrium trihydride under hydrostatic pressure. *Solid State Commun.* **138**, 436 – 440 (2006).

S3. Palasyuk, T. & Tkacz, M. Hexagonal to cubic phase transition in $YH_3$ under high pressure. *Solid State Commun.* **133**, 477 – 480 (2005).

S4. Pressure as a function of volume at a given temperature $T$ is written as $P_T = 3K_0(\frac{V}{V_0})^{-2/3}[1 - (\frac{V}{V_0})^{1/3}]\exp\{\frac{3}{2}(K_0' - 1)[1 - (\frac{V}{V_0})^{1/3}]\}$; where $K_0$ is a bulk modulus, $K_0' = \partial K_0/\partial P$. Vinet, P., Ferrante, J., Smith, J.R. & Rose, J.H. A universal equation of state for solids. *J. Phys. C: Solid State Phys.* **19**, L467–L473 (1986).

S5. Joly, Y. X-ray absorption near-edge structure calculations beyond the muffin-tin approximation. *Phys. Rev. B* **63**, 125120 (2001).

S6. Bunău, O. & Joly, Y. Self-consistent aspects of x-ray absorption calculations. *J. Phys.: Condens. Matter* **21**, 345501 (2009).

S7. Hedin, L. & Lundqvist, B.I. Explicit local exchange-correlation potentials. *J. Phys. C: Solid State Phys.* **4**, 2064–2083 (1971).

S8. Ravel, B. & Newville, M. *ATHENA*, *ARTEMIS*, *HEPHAESTUS*: data analysis for X-ray absorption spectroscopy using *IFEFFIT*. *J. Synchrotron Radiat.* **12**, 537–541 (2005).

S9. Timoshenko, J., Kuzmin, A. & Purans, J. EXAFS study of hydrogen intercalation into $ReO_3$ using the evolutionary algorithm. *J. Phys.: Condens. Matter* **26**, 055401 (2014).

S10. Udovic, T., Huang, Q. & Rush, J. Characterization of the structure of $YD_3$ by neutron powder diffraction. *J. Phys. Chem. Solids* **57**, 423–435 (1996).

S11. Ankudinov, A.L., Ravel, B., Rehr, J.J. & Conradson, S.D. Real-space multiple-scattering calculation and interpretation of x-ray-absorption near-edge structure. *Phys. Rev. B* **58**, 7565–7576 (1998).

S12. Timoshenko, J. & Kuzmin, A. Wavelet data analysis of EXAFS spectra. *Comput. Phys. Commun.* **180**, 920–925 (2009).

S13. Klementev, K.V. Extraction of the fine structure from x–ray absorption spectra. *J. Phys. D: Appl. Phys.* **34**, 209–217 (2001).
